# The Effectiveness and Feasibility of Conversational Agents in Supporting Care for Patients With Cancer: Systematic Review and Meta-Analysis

**DOI:** 10.2196/76968

**Published:** 2025-08-08

**Authors:** Xiao-han Jiang, Xiu-hong Yuan, Hui Zhao, Jun-sheng Peng

**Affiliations:** 1 School of Nursing Sun Yat-sen University Guangzhou China; 2 Department of Gastric Surgery Collaborative Innovation Center for Cancer Medicine Sun Yat-sen University Cancer Center Guangzhou China; 3 Department of Gastric Surgery Guangdong Provincial Key Laboratory of Colorectal and Pelvic Floor Diseases Sixth Affiliated Hospital of Sun Yat-sen University Guangzhou China

**Keywords:** conversational agent, conversational agents, artificial intelligence, cancer, care, systematic review, meta-analysis

## Abstract

**Background:**

Patients with cancer experience complex physical, psychosocial, and behavioral challenges that require continuous support. This need has intensified with the rising cancer burden worldwide and the limited scalability of traditional care models. In response, conversational agents (CAs) have emerged as promising digital interventions for enhancing cancer care, but evidence regarding their feasibility and effectiveness remains limited.

**Objective:**

This study aimed to evaluate the feasibility and effectiveness of CAs in supporting care for patients with cancer and to summarize the key characteristics of CA interventions to inform future design and implementation.

**Methods:**

We systematically searched PubMed, Cochrane Library, Web of Science, and Embase databases from the index date through February 3, 2025, and screened reference lists and trial registries for gray literature. Eligible studies included randomized controlled trials (RCTs) and nonrandomized interventions (NRIs) evaluating CA-delivered interventions targeting health outcomes in patients with cancer. Two reviewers independently selected studies and extracted data. Study quality was then appraised using the Cochrane Risk of Bias 2.0 tool for RCTs and the Joanna Briggs Institute (JBI) Critical Appraisal Checklist for NRIs. Extracted data included study characteristics, CA features, and implementation outcomes, including feasibility, acceptability, and usability. Meta-analyses were conducted on physical activity, pain, anxiety, depression, psychological distress, and quality of life. Narrative synthesis was used for outcomes with inconsistent reporting across studies, including health information acquisition and treatment-related side effects.

**Results:**

In total, 17 studies involving 1817 patients with cancer were included, with 10 (58.8%) studies being included in the meta-analysis. The meta-analysis showed significant improvements in physical activity (mean difference [MD]=1.44, 95% CI 0.36-2.52, *P*<.01), pain (MD=–0.91, 95% CI –1.44 to –0.38, *P*<.01), anxiety (SMD=–0.19, 95% CI –0.35 to –0.02, *P*=.02), and quality of life (SMD=0.35, 95% CI 0.03-0.67, *P*=.03). No significant effects were observed on depression (SMD=–0.07, 95% CI –0.42 to 0.27, *P*=.68) or psychological distress (SMD=–0.33, 95% CI –0.66 to 0.01, *P*=.06). Narrative synthesis suggested that CAs have the potential to improve patients’ acquisition of health information and help manage treatment-related side effects. Notably, CAs were generally found to be safe, feasible, acceptable, and usable among patients with cancer, particularly during the initial phase of use. However, user engagement tended to decline over time, underscoring the need for strategies to sustain long-term use.

**Conclusions:**

This systematic review is the first comprehensive analysis to suggest that CAs are feasible, acceptable, usable, and effective interventions for patients with cancer. Nevertheless, the limited psychological benefits and suboptimal long-term user engagement indicate the need for further refinement. Future research should adopt theory-based designs and leverage emerging technologies to enhance personalization, empathy, and sustained engagement in CA interventions. Robust evidence from large-scale RCTs is needed to strengthen the evidence base.

**Trial Registration:**

PROSPERO CRD42025645982; https://www.crd.york.ac.uk/PROSPERO/view/CRD42025645982

## Introduction

### Background

Cancer remains a leading cause of morbidity and mortality worldwide, placing a substantial burden on health care systems. In 2022, an estimated 20 million new cancer cases and 9.7 million cancer-related deaths occurred worldwide, with approximately 53.5 million individuals living with cancer [1]. A cancer diagnosis not only presents significant challenges to health care systems but also profoundly affects patients’ daily lives. Both cancer itself and its treatment can result in a wide range of physical symptoms and psychological distress [2]. These multifaceted burdens frequently disrupt patients’ overall well-being and significantly impair their quality of life [2].

In this context, there has been increasing emphasis on developing effective cancer care that provides continuous, comprehensive support alongside standard oncological treatment, aiming to address patients’ physical and psychological needs and to promote behavioral change across the cancer care continuum [3]. However, implementing such care models through traditional face-to-face delivery remains difficult, as these approaches are often constrained by time, staffing, and scalability—particularly in resource-limited settings [4]. As the demand for personalized and continuous support grows and health care systems shift toward cost-efficient care, these limitations have become increasingly evident, highlighting the necessity for automated, scalable, and cost-effective solutions to enhance care for patients with cancer [5].

In response to these limitations, there has been growing interest in leveraging mobile health (mHealth) technologies to enhance care delivery. In particular, conversational agents (CAs) have emerged as a promising mHealth innovation in supporting care for patients with cancer [6]. CAs are dialogue systems capable of understanding and generating human language to enable effective human-computer interactions [7]. Unlike conventional mHealth tools—which often rely on static content, unidirectional reminders, or resource-intensive human support—CAs provide automated, real-time dialogue capabilities that support dynamic, personalized interactions [8]. By simulating human-like conversations, CAs can adapt to patients’ evolving informational, emotional, and practical needs—an essential feature in the complex and often unpredictable trajectory of cancer care. These capabilities may not only improve the continuity and personalization of care but also offer a scalable means of support, potentially reducing the burden on health care providers [9].

Although CA interventions have shown promise and are increasingly used in cancer care, evidence regarding their feasibility and effectiveness remains inconclusive [10]. Although some studies have reported positive outcomes—such as improved symptom management, psychological support, behavior change, and quality of life—others have shown minimal or no effects [11,12]. These inconsistencies highlight the need for a comprehensive synthesis of existing evidence to clarify the effectiveness of CAs in supporting cancer care [10]. In parallel, although feasibility is frequently cited as a strength of CA interventions—due to their ability to deliver personalized support, reduce access barriers, and provide timely responses—several concerns have also emerged, including risks of miscommunication, limited empathetic responsiveness, and challenges in sustaining use [13,14]. Together, these inconsistencies in both clinical and implementation outcomes point to the urgent need for a systematic synthesis to guide the design and integration of CA interventions in cancer care.

To date, no systematic review has comprehensively evaluated the effectiveness or feasibility of CA-based interventions in cancer care. Existing reviews remain limited in scope; notably, only 1 previous narrative review provides a broad overview of CA applications in oncology but does not quantitatively synthesize outcomes or systematically synthesize implementation outcomes [10]. Consequently, it remains unclear whether CAs lead to improvements in health outcomes of patients with cancer; what implementation-related patterns—particularly regarding feasibility, usability, and acceptability—have emerged; and what core features define current CA interventions for cancer care. A rigorous synthesis of the existing evidence is warranted to inform future CA development, facilitate clinical implementation, and support evidence-based decision-making.

### Objectives

This systematic review and meta-analysis aimed to comprehensively evaluate the use of CA interventions in cancer care. Specifically, the objectives were to (1) assess their effectiveness in improving patient outcomes, including quality of life, pain, anxiety, depression, psychological distress, physical activity, treatment-related side effects, and health information acquisition; (2) evaluate implementation outcomes—such as feasibility, acceptability, and usability—to determine the practical applicability of CAs in oncology settings; and (3) synthesize the core characteristics of CA interventions, including intervention components, and CA delivery and interaction logic, as well as input and output modalities, to inform future development and implementation.

## Methods

### Study Protocol and Registration

This systematic review and meta-analysis was based on a prespecified protocol registered with PROSPERO (International Prospective Register of Systematic Reviews; CRD42025645982) and conducted and reported according to the PRISMA (Preferred Reporting Items for Systematic Reviews and Meta-Analyses) statement [15]. The PRISMA checklist can be found in in Table S1 in Multimedia Appendix 1.

### Search Strategy

A comprehensive systematic literature search was conducted on 4 different electronic databases—PubMed, Embase, Web of Science, and Cochrane Library—from the index date through February 3, 2025. After study screening and selection, references for the identified studies and relevant websites, such as clinical trial registries, were manually searched to find potential related gray papers. The keywords of the search mainly revolved around “cancer” and “conversational agent.”. The full search strategies and the number of search results from each database are detailed in Table S2 in Multimedia Appendix 1.

### Inclusion and Exclusion Criteria

We considered eligible randomized controlled trials (RCTs) and nonrandomized interventions (NRIs) that examined the effects of CAs, defined as dialogue systems capable of understanding and generating human language to enable effective human-computer interactions. Eligible studies aimed to improve health-related outcomes in adult patients with cancer using CAs that were either specifically developed for oncology care or originally designed for general health purposes and subsequently applied to patients with cancer. The inclusion and exclusion criteria are reported in Textbox 1.

**Inclusion and exclusion criteria for screening papers.**

Inclusion criteria:

- Population: participants aged ≥18 years, of both sexes, diagnosed with cancer
- Intervention: online interventions delivered through conversational agents (CAs), which were defined as dialogue systems capable of understanding and generating human language to enable effective human-computer interactions
- Comparison: any comparator was acceptable (no intervention, usual care, active comparators, or a within-subject pre-post design)
- Outcomes: studies reporting health-related outcomes of patients with cancer
- Study design: studies reporting original data from randomized controlled trials (RCTs) and nonrandomized interventions (NRIs)

Exclusion criteria:

- Duplicate papers
- Literature for which full text is not available, such as conference abstracts
- Unrelated to the topic

### Screening and Study Selection

All studies identified through database searches, reference lists, and relevant websites were imported into EndNote X9 (Clarivate Analytics), a reference management software program. EndNote was also used to detect and remove any duplicate entries. The selection of studies was conducted in 2 stages. Initially, 2 independent reviewers (authors JXH and YXH) screened the titles and abstracts of all identified studies in EndNote to exclude studies that did not meet the basic inclusion criteria. Subsequently, the full texts of the remaining studies were retrieved and assessed in detail against the inclusion and exclusion criteria by the same 2 reviewers. Any disagreements between reviewers were resolved through discussion or, if necessary, consultation with a third reviewer (author PJS).

### Data Extraction

Data extraction using Microsoft Excel was first pilot-tested on 3 studies and revised with additional headings before being applied to all included studies. Two independent reviewers (JXH and YXH) conducted the data extraction process, with any disagreements resolved by a third reviewer (PJS). The following data were extracted: (1) basic study characteristics (author, title, country, and publication year), (2) sample characteristics (cancer type, sample size, age, and gender), (3) intervention components (consultable content, CA-only vs multicomponent interventions, frequency and duration, theoretical framework), (4) CA delivery and interaction logic (CA name, delivery platform, prompt sequence, whether the CA was cancer specific, clinician referral, algorithm type, and embodiment type), (5) input and output information (input data and format, input and output modality, and output source), and (6) outcome measures (health outcomes, feasibility, acceptability, and usability).

### Quality Evaluation

Two independent authors (JXH and YXH) assessed the risk of bias for included studies using the revised Cochrane Risk of Bias Tool version 2.0 (ROB 2.0) for RCTs [16] and the Joanna Briggs Institute (JBI) Critical Appraisal Checklist for NRIs [17]. When disagreements arose, a third reviewer (PJS) was consulted, and consensus was achieved through discussion.

### Statistical Methods

The primary outcome was quality of life, selected for its direct relevance in assessing the overall effectiveness of CA interventions in cancer care. Secondary outcomes included effectiveness outcomes (physical activity, pain, anxiety, depression, psychological distress, treatment-related side effects, and health information acquisition) and implementation outcomes (feasibility, acceptability, and usability). Depending on data availability and consistency, either a meta-analysis or a narrative synthesis was conducted.

Effectiveness outcomes were synthesized using meta-analysis when the included studies demonstrated sufficient statistical homogeneity. Analyses were performed using Review Manager (RevMan) version 5.4 (Cochrane Collaboration). For each outcome, either change-from-baseline scores or postintervention scores were extracted, with a consistent approach was applied within each analysis. When necessary, missing summary statistics were estimated following the methods recommended in the *Cochrane Handbook for Systematic Reviews of Interventions* [18]. Effect sizes were reported as standardized mean differences (SMDs) with 95% CIs when different measurement scales were used, and as mean differences (MDs) when the same scale was applied. Heterogeneity was assessed using the chi-square test and the *I*² statistic. If *P*>.10 and *I*^2^ < 50%, it suggested that the heterogeneity among studies as small, and the fixed-effect model was selected. If *P*<.10 and *I*^2^> 50%, it suggested that there was obvious heterogeneity among the studies, and the random-effects model was adopted [19]. Sensitivity analysis was performed by comparing the results of the combined effect sizes of the fixed-effects model and random-effects model to ensure stability and reliability of the results [18]. Publication bias was examined using funnel plots.

For effectiveness outcomes not amenable to meta-analysis due to insufficient data or heterogeneity, a narrative synthesis was conducted. This synthesis was structured by outcome domain and described the direction and magnitude of reported effects. For implementation outcomes, a narrative synthesis was conducted and organized thematically according to key evaluation dimensions, including feasibility (retention rate, safety, interactions, duration of engagement, and engagement rate), acceptability (satisfaction, perceived helpfulness, and recommendation willingness), and usability (usability of content, ease of use, and user experience). In addition, intervention characteristics—including intervention components, CA delivery and interaction logic, and input and output information—were descriptively summarized.

## Results

### Results of Literature Search

**Figure 1:**
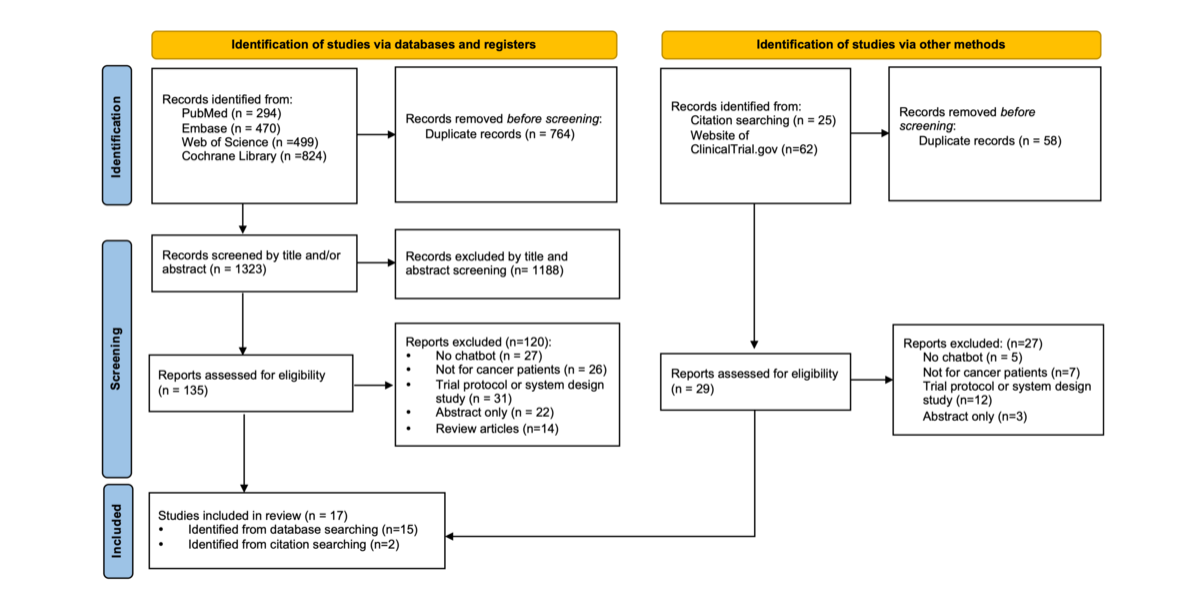
PRISMA flow diagram showing the study selection process. PRISMA: Preferred Reporting Items for Systematic Reviews and Meta-Analyses.

The database search yielded 2087 results. A total of 1323 (63.4%) titles and abstracts were screened after removal of duplicates, and 1188 (56.9%) studies were excluded after screening. In total, 135 (6.5%) potential full-text papers were identified for further evaluation, of which 15 (11.1%) met our inclusion criteria. An additional 87 papers were identified from websites and relevant citations. After removing duplicates and assessing titles, abstracts, and full texts, 2 extra papers were identified and included. In total, 17 papers were included in this systematic review (Figure 1).

### Study and Sample Characteristics

The 17 included studies [6,11,12,20-34] were published between 2016 and 2024 (please note that Bema [27] contains the complete documentation of results from Mlakar et al’s [26] study). There were a total of 1817 participants, and sample sizes ranged between 30 (1.7%) and 336 (18.5%). The mean age of the participants ranged between 25 (SD 2.9) and 64 (SD 11.8) years. Of the 17 studies, 12 (70.6%) were RCTs, and 5 (29.4%) were NRIs. Most studies were conducted in the United States (n=10, 58.8%) and primarily targeted patients with breast cancer (n=8, 47.1%). Overall, 69.7% (n=1266) of the participants were women. A summary of study characteristics is presented in Table 1.

**Table 1:** Basic information about the included papers (N=17).

Study; country	Research type	Sample size (N) and participants	Age (years), mean (SD)	Female gender, n (%)	Consultable content	Outcomes
Baumgärtner et al [6]; Germany	2-arm RCT^a^	112 patients with prostate cancer	64.0 (11.8)	0	General prostate cancer management	User health, feasibility, acceptability, and usability data
Tawfik et al [11]; Egypt	3-arm RCT	150 patients with breast cancer	44.7 (7.0)	150 (100.0)	Symptom management	User health and usability data
Greer et al [12]; United States	2-arm RCT	45 patients with cancer	25.0 (2.9)	36 (80.0)	Psychological management	User health, feasibility, acceptability, and usability data
Schmitz et al [20]; United States	2-arm RCT	36 patients with breast cancer	53.4 (11.0)	36 (100.0)	Symptom management	User health, feasibility, acceptability, and usability data
Sikorskii et al [21]; United States	2-arm RCT	272 patients with cancer	61.0 (12.0)	69 (51.0)	Symptom management and medication reminders	User health data
Hassoon et al [22]; United States	3-arm RCT	42 patients with cancer	62.1 (9.8)	38 (90.0)	Physical activity management	User health and feasibility data
Maguire et al [23]; United Kingdom	2-arm RCT	336 patients with cancer	52.4 (12.2)	275 (81.8)	Symptom management	User health and feasibility data
Lee et al [24]; Korea	4-arm RCT	145 patients with breast cancer	—^b^	145 (100.0)	Management regarding radiotherapy	User health and feasibility data
Horesh et al [25]; United States	NRI^c^	37 patients with breast and ovarian cancer	46.8 (6.8)	37 (100.0)	Psychological management	User health and acceptability data
Mlakar et al [26,27]; Slovenia	NRI	160 patients with breast and colorectal cancer	55.0 (—)	129 (77.7)	General cancer management	User health data
Bibault et al [28]; France	2-arm RCT	142 patients with breast cancer	42.0 (19.0)	142 (100.0)	General breast cancer management	User health data
Al-Hilli et al [29]; United States	2-arm RCT	37 patients with breast cancer	62.0 (13.0)	37 (100.0)	Genetic counseling management	User health and acceptability data
Kamdar et al [30]; United States	2-arm RCT	112 patients with cancer	52.7 (8.5)	55 (49.2)	Pain management	User health, feasibility, and usability data
Queiroz et al [31]; Brazil	NRI	30 patients with colorectal cancer	50.3 (13.4)	13 (43.3）	General colorectal cancer management	User health, feasibility, and usability data
Bickmore et al [32]; United States	2-arm RCT	89 patients with cancer	59.2 (9.8)	48 (46.0)	Clinical trial information	User health and acceptability data
Gomaa et al [33]; United States	NRI	34 patients with gastrointestinal cancer	61.0 (12.0)	19 (56.0)	Symptom management	User health, feasibility, acceptability, and usability data
Caru et al [34]; United States	NRI	38 patients with breast cancer	52.4 (11.0)	38 (100.0)	General breast cancer management	User health data

^a^RCT: randomized controlled trial.

^b^Not available.

^c^NRI: nonrandomized intervention.

### Characteristics of CA Interventions

The characteristics of the CA interventions are presented in Table S3 in Multimedia Appendix 1.

#### Intervention Components

In terms of consultable content, most CAs delivered general management targeting specific malignancies (n=6, 35.3%), including breast cancer [20,26,28,34], colorectal cancer [31], and prostate cancer [6]. Several other studies (n=5, 29.4%) provided symptom management [11,21,23,30,33], occasionally incorporating medication reminders [21] or addressing specific pain symptoms [30]. Two studies provided psychological management [12,25]. Individual studies focused on radiotherapy-related management [24], physical activity [22], genetic counseling [29], or clinical trial information [32].

Regarding intervention components, 8 (47.1%) studies used CA-only interventions [6,11,12,21,28,29,32,33], while 9 (52.9%) studies integrated additional interventions, such as mHealth apps [26], medical team referrals [20,23,24,30,31,34], printed education materials [24], mindfulness therapy [25,34], wearable sensors [22,31], and prescription refill request tools [30]. Intervention durations ranged from 4 weeks to 6 months, except for 3 (17.6%) studies that adopted a single-session design.

Most studies did not explicitly adopt a theoretical framework. Only 3 (17.6%) studies reported using theoretical frameworks: one used the Empowerment Education Model [11]; another applied stress and coping theory, together with the broaden-and-build theory of positive emotion [12]; and the third adopted cognitive behavioral therapy as its theoretical basis [25]; see Table S3 in Multimedia Appendix 1.

#### CA Delivery and Interaction Logic

Across the 17 studies, 17 CAs were deployed, with 2 (11.8%) studies using the same CA (Nurse Amie) [20,34] and 1 (5.9%) study [22] incorporating 2 CAs (MyCoach and CoachText) [22]. These CAs operated through various digital platforms, including custom-developed mHealth apps (n=5, 29.4%) [23,25,26,30,32], Facebook Messenger (n=3, 17.6%) [12,28,31], SMS (n=3, 17.6%) [22,29,33], Amazon Echo Show and Alexa (n=2, 11.8%) [20,22], Kakao (n=1, 5.9%) [24], phone calls (n=1, 5.9%) [21], Microsoft Azure (n=1, 5.9%) [11], and a website (n=1, 5.9%) [6]. Of the 17 included studies, 14 (82.4%) evaluated CAs specifically designed for patients with cancer [6,11,20-24,26,28,30-34], while the remaining 3 (17.6%) involved CAs originally developed for general health purposes, such as managing chronic conditions or delivering preventive health counseling, but were applied to patients with cancer in the study context [12,25,29].

The sequencing of prompts to the CAs generally followed 3 distinct modes: user-initiated inputs, where users actively started the interaction (n=4, 23.5%) [11,22,24,28]; system-initiated prompts, where messages were proactively delivered by the CAs based on a predefined schedule or contextual triggers, which do not require prior user input but allow for subsequent user interaction (n=4, 23.5%) [12,21,26,32]; and hybrid approaches that combined both user- and system-initiated interactions (n=9, 52.9%) [6,20,23,25,29-31,33,34].

In terms of clinician referral, only 6 (35.3%) studies incorporated mechanisms to hand over user inquiries to health care professionals, when necessary [20,23,24,30,31,34], while 11 (64.7%) did not [6,11,12,21,22,25,26,28,29,32,33].

In terms of embodiment, 5 (29.4%) CAs were virtual, using visual avatars or animated characters [20,22,25,26,32], while the remaining 12 (70.6%) were disembodied [6,11,12,21-24,28-31,33], interacting without visual representation.

Additionally, CAs used 3 types of dialogue approaches: 5 (29.4%) used artificial intelligence (AI)-based methods (leveraging AI techniques such as machine learning and natural language processing to produce contextually relevant responses) [20,22,26,28,34], 5 (29.4%) used a rule-based approach (generating deterministic responses through predefined rules and structured pathways) [11,12,21,23,33], and 7 (41.2%) adopted a hybrid dialogue approach (integrating both AI- and rule-based logic) [6,24,25,29-32]; see Table S3 in Multimedia Appendix 1.

#### Input and Output Information

Input data included symptoms, adverse effects, adherence behaviors (medication and treatment), lifestyle behaviors (diet, nutrition, physical activity), emotions, treatment procedures, therapy feedback and planning, genetic counseling, clinical trial information, diagnostic information, and appointment scheduling. Users interacted with CAs through 3 distinct input methods: free input, where users entered open-ended queries (n=3, 17.6%) [11,22,28]; button input, involving the selection of predefined topics or numerical options (n=6, 35.3%) [21-23,25,32,33]; and mixed input, which combined both free and button-based interactions (n=8, 47.1%) [6,12,20,24,26,29-31].

Output information was predominantly text based (n=9, 52.9%) [6,11,22,25,28-31,33], followed by multimodal formats (eg, audio and video; n=5, 29.4%) [12,23,24,26,32] and voice-based outputs (n=3, 17.6%) [11,20,22]. The content delivered by CAs was generally derived from authoritative sources. These included clinical guidelines from organizations such as the American Cancer Society (ACS), National Cancer Institute (NCI), and the National Comprehensive Cancer Network (NCCN); peer-reviewed literature; expert consensus; evidence-based question-and-answer (QnA) libraries developed by study teams; and authoritative health websites [12,21,23,25,26,28-33]. Additionally, 6 (35.3%) studies used cloud-based platforms, such as Amazon Alexa, Microsoft QnA Maker, SAP Conversational AI, and Kakao chatbot frameworks, to retrieve responses from predefined, evidence-informed knowledge bases [6,11,20,22,24,34]; see Table S3 in Multimedia Appendix 1.

### Feasibility, Acceptability, and Usability of CAs

The studies included in the qualitative synthesis of feasibility, acceptability, and usability are listed in Table S4 in Multimedia Appendix 1.

#### Feasibility

Feasibility outcomes of CA interventions were reported in 9 (52.9%) of the 17 included studies, focusing primarily on (1) safety, (2) retention rate, (3) engagement rate, (4) interactions, and (5) duration of engagement [6,12,20,22-24,30,31,33]. Safety was assessed in 3 (33.3%) studies, all of which reported no adverse events, indicating that CAs are generally safe for participants [20,22,23]. Retention rates—defined as the proportion of participants who completed postintervention assessments—were reported in 7 (63%) studies and ranged from 64% to 85.7%, with most exceeding 70% [6,12,20,23,30,31,33]. Engagement rates—defined as the proportion of participants who interacted with the CA at least once during the intervention—were reported in 4 (44.4%) studies and ranged from 59% to 86% [6,30,31,33]. The interaction frequency varied considerably, with participants engaging with the CA between 3 and 15 times on average and usage frequencies ranging from 2.1 times per week to twice daily [6,12,22,24,30,33]. Two studies reported usage duration metrics: one study documented a mean total usage time of 73.8 (SD 52) minutes over 4 weeks [12], while another reported an average of 3.4 usage days per participant within the same period [6]. Notably, 3 (33.3%) studies observed a declining trend in engagement over time, including reductions in the interaction frequency and the proportion of active users [30,31,33]. Overall, CA interventions are feasible and generally safe in patients with cancer, with promising levels of initial engagement. However, maintaining sustained user involvement remains a significant challenge that warrants attention in future design and implementation efforts.

#### Acceptability

Of the 17 studies, 7 (41.2%) assessed acceptability, focusing on (1) satisfaction, (2) perceived helpfulness, and (3) recommendation willingness. Satisfaction with CA interventions was consistently high, with rates ranging from 70% to 97% in 4 (57.1%) studies [12,20,25,29], and 1 (14.3%) study [29] reporting comparable satisfaction to in-person consultations and higher ratings than traditional digital interfaces. Perceived helpfulness, evaluated in 5 (71.4%) studies, was generally favorable, with 70%-90.7% of participants finding the interventions beneficial in 4 (80%) studies [6,12,25,33]; in 1 (20%) study, participants rated the interventions 5.76 out of 10 in terms of perceived help with symptom relief, indicating a moderate level of perceived helpfulness in addressing symptoms [20]. Recommendation willingness was evaluated in 4 (57.1%) studies, with scores ranging from 6.9 to 7.24 out of 10, and 95.3%-97% of participants agreed to recommend CAs for broader clinical implementation [6,12,20,25]. Collectively, these findings suggest that CA interventions are generally well accepted by patients with cancer.

#### Usability

Usability of CAs was evaluated in 7 (41.2%) of the 17 studies, focusing on 3 key aspects: (1) usability of content, (2) ease of use, and (3) user experience. Usability of content reported in 5 (71.4%) studies received consistently high scores ranging from 79.6 to 86.14—well above the 68-point threshold for acceptable usability [20,30,31,33]. Additionally, in 1 (14.3%) study, 94% of participants described CA responses as useful and informative [11]. Ease of use, assessed in 3 (42.9%) studies, was also highly rated, with 94%-100% of participants agreeing that the CAs were easy to use [6,11,30]. User experience reported in 3 (42.9%) studies was generally positive [11,31,33]. Participants highlighted several valued features of the CAs, including their nonjudgmental nature, intuitive navigation, effective error management, decision-making support, personalized educational content, motivational messaging, and communication skill support in clinical interactions [11,12,31,33].

### Effectiveness of CA Interventions: Meta-Analysis

**Figure 2:**
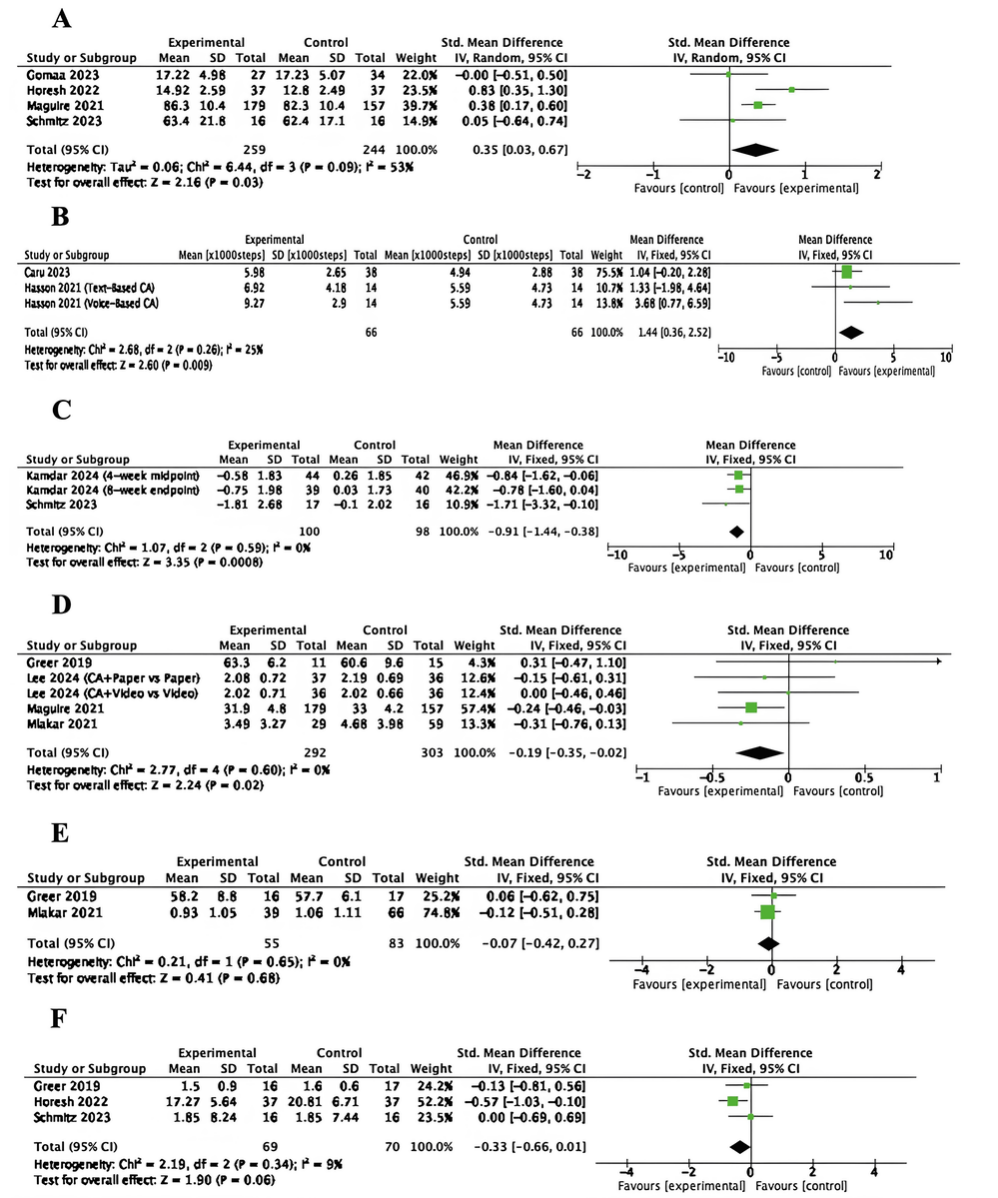
Forest plot of (A) quality of life, (B) physical activity (×1,000 steps), (C) pain, (D) anxiety, (E) depression, and (F) psychological distress.

Of the 17 studies, 10 (58.8%) were included in a meta-analysis, comprising 6 (60%) RCTs [12,20,22-24,30] and 4 (40%) NRIs [25,26,33,34]. Given the clinical and methodological similarities across the included studies, quality of life, physical activity, pain, anxiety, depression, and psychological distress were adopted as outcome indicators of the meta-analysis. The results of the meta-analysis are presented in Table 2 and Figure 2. Sensitivity analyses comparing fixed- and random-effects models for each of these outcomes yielded consistent results, supporting the stability of the pooled effect estimates (see Figures S1-S6 in Multimedia Appendix 1).

**Table 2:** Summary of pooled effects of CA^a^ interventions across health outcomes.

Outcome	Patients (N=1817), n (%)	Studies (N=17), n (%)	Effect size (95% CI)	*P* value	Statistical heterogeneity, *P* value, *I*²	Effect model
Quality of life	503 (27.7)	4 (23.5) [23,25-27,33]	SMD^b^=0.35 (0.03 to 0.67)	.03	.09, 53%	Random
Physical activity	118 (6.5)	2 (11.8), 3 comparisons [22,34]	MD^c^=1.44 (0.36 to 2.52)	<.01	.26, 25%	Fixed
Pain	120 (6.6)	2 (11.8), 3 comparisons [20,30]	MD=–0.91 (–1.44 to –0.38)	<.01	.59, 0%	Fixed
Anxiety	595 (32.7)	4 (23.5), 5 comparisons [12,23,24,26,27]	SMD=–0.19 (–0.35 to –0.02)	.02	.60, 0%	Fixed
Depression	138 (7.6)	2 (11.8) [12,26,27]	SMD=–0.07 (–0.42 to 0.27)	.68	.65, 0%	Fixed
Psychological distress	139 (7.6)	3 (17.6) [12,20,25]	SMD=–0.33 (–0.66 to 0.01)	.06	.34, 9%	Fixed

^a^CA: conversational agent.

^b^SMD: standardized mean difference.

^c^MD: mean difference.

#### Quality of Life

Quality of life was measured in 4 (23.5%) studies involving 503 (27.7%) participants (Figure 2A and Table 2) [23,25,26,33]. Moderate heterogeneity was observed among the included studies (*I*²=53%, *P*=.09), supporting the use of a random-effects model. The pooled analysis showed statistically significant improvement in quality of life in the intervention group compared to the control group (standardized mean difference [SMD]=0.35, 95% CI 0.03-0.67, *P*=.03).

#### Physical Activity

Physical activity was measured using the step count in 2 (11.8%) studies involving 118 (6.5%) participants (Figure 2B and Table 2) [22,34]. One study with a 3-arm design contributed 2 independent comparisons: a text-based CA intervention and a voice-based CA intervention, each compared with the usual care group. The pooled analysis demonstrated a statistically significant improvement in physical activity in the intervention group compared to the control group (mean difference [MD]=1.44, 95% CI 0.36-2.52, *P*<.01). These studies showed low heterogeneity (*I*^2^=25%, *P*=.26).

#### Pain

Pain was measured in 2 (11.8%) studies involving 120 (6.6%) participants [20,30]. In 1 (50%) study, outcomes at 2 timepoints were included as separate entries in the meta-analysis, with assessments conducted at the midpoint (4 weeks) and the endpoint (8 weeks) of the intervention [30]. The results indicated no significant heterogeneity among the included studies (*I*^2^=0%, *P*=.59), supporting the use of a fixed-effects model. The pooled analysis demonstrated a statistically significant reduction in pain scores in the intervention group compared to the control group (MD=–0.91, 95% CI –1.44 to –0.38, *P*<.01). These findings suggest that the CA intervention was effective in reducing the pain of patients with cancer (Figure 2C and Table 2).

#### Anxiety

Anxiety was assessed in 4 (23.5%) studies involving a total of 595 (32.7%) participants [12,23,24,26]. One study with a 4-arm design contributed 2 independent comparisons: CA + paper versus paper, and CA + video versus video [24]. The meta-analysis showed no heterogeneity among the included trials (*I*²=0%, *P*=.60), supporting the use of a fixed-effects model. The pooled results demonstrated a statistically significant reduction in anxiety levels in the intervention group compared to the control group (SMD=–0.19, 95% CI –0.35 to –0.02, *P*=.02). These findings suggest that the CA intervention was effective in alleviating anxiety (Figure 2D and Table 2).

#### Depression

Depression was measured in 2 (11.8%) studies involving 138 (7.6%) participants (Figure 2E and Table 2) [12,26]. The pooled analysis demonstrated a nonsignificant SMD of –0.07 (95% CI –0.42 to 0.27, *P*=.68), with no heterogeneity among the included trials (*I*²=0%, *P*=.65).

#### Psychological Distress

Psychological distress was measured in 3 (17.6%) studies involving 139 (7.6%) participants (Figure 2F and Table 2) [12,20,25]. The pooled analysis demonstrated a nonsignificant SMD of –0.33 (95% CI –0.66 to 0.01, *P*=.06), with low heterogeneity among the included trials (*I*²=9%, *P*=.34).

### Effectiveness of CA Interventions: Narrative Synthesis

#### Health Information Acquisition

Health information acquisition was reported in 4 (57.1%) of the 17 studies, all of which used short-term designs and collectively included all 3 single-session interventions identified in this review. Due to heterogeneity in outcome measures, a meta-analysis was not feasible; therefore, a narrative synthesis was conducted. Across these studies, Bickmore et al [32] found that CAs enhance access to clinical trial information among patients with cancer with limited health literacy. Bibault et al [28] and Al-Hilli et al [29] further demonstrated that CAs effectively enhance patients’ understanding of clinical information by providing accurate and specific content. Baumgärtner et al [6] reported a significant reduction in unmet informational needs among patients receiving CA intervention. Despite their short duration, these CA interventions demonstrated the potential to effectively improve patients’ acquisition of health information.

#### Treatment-Related Side Effects

Of the 17 studies, 3 (17.6%) examined CA interventions designed to support patients in managing side effects associated with cancer treatments, including oral oncolytic agents, chemotherapy, and surgery. Due to heterogeneity in outcome measures and definitions, a narrative synthesis was conducted. Queiroz et al [31] found that CAs facilitate effective self-monitoring of treatment-related side effects. Tawfik et al [11] reported improvements in self-management capacity for chemotherapy-induced symptoms. Sikorskii et al [21] further demonstrated a notable reduction in symptom severity among patients taking oral oncolytic agents. These findings indicate that CAs may serve as a valuable tool to empower patients in alleviating side effects throughout the cancer care continuum.

### Summary of Quality Assessment and Risk of Bias

The results of risk-of-bias assessments of the 17 studies are reported in Figures S7 and S8 and Table S5 in Multimedia Appendix 1. Of the 12 (70.6%) RCTs, 3 (25%) had an overall low risk of bias [21-23], 4 (33.3%) exhibited a high risk of bias [6,12,20,32], and another 5 (41.7%) raised some concerns [11,24,28-30]. Common methodological issues included inadequate randomization, deviations from intended interventions, and bias due to missing outcome data or outcome measurement. In addition, 5 (41.7%) quasi-experimental studies met 7-8 out of 9 quality assessment criteria, indicating relatively good methodological quality [25,26,31,33,34]. Common limitations among these studies included the absence of control groups, limited follow-up reporting, and insufficient pre- and postintervention outcome measurement.

## Discussion

### Principal Findings

This systematic review is the first to comprehensively evaluate the effectiveness and feasibility of CA interventions in supporting care for patients with cancer. In total, 17 trials involving 1817 participants were included, comprising 12 (70.6%) RCTs and 5 (29.4%) NRIs. Of these, 10 (58.8%) studies were included in a meta-analysis. The meta-analysis findings indicated that CA interventions are effective in promoting physical activity, reducing pain and anxiety, and improving the quality of life among patients with cancer. However, no significant effects were observed for depression or psychological distress. Narrative synthesis suggested that CAs have the potential to enhance health information acquisition and help manage treatment-related side effects. Furthermore, CAs were generally found to be feasible, acceptable, and usable among patients with cancer, particularly during the initial phase of use. However, user engagement tended to decline over time, underscoring the need for strategies to sustain long-term use. These results support the potential integration of CAs into broader cancer care frameworks and inform future direction for optimizing CA design to enhance sustained engagement and clinical effectiveness.

### Comparison With Prior Work

Prompt sequencing and interaction modes are central to the design and delivery of CA interventions, shaping how users engage with the CA system and receive tailored support. Across the included studies, CAs adopted user-initiated inputs [11,22,24,28], system-initiated prompts [12,21,26,32], or a combination of both approaches [6,20,23,25,29-31,33,34]. The sequencing of prompts generally aligned with the CA’s intended function and interaction style: user-initiated prompts supported on-demand health queries or symptom check-ins; system-initiated prompts provided scheduled reminders or symptom-triggered alerts; and hybrid models combined both to enhance adherence and engagement [8]. Upon receiving input, CAs typically conducted a needs assessment and generated personalized responses via rule-based scripts [11,12,21,23,33], AI-driven dialogue generation [20,22,26,28,34], or both approaches [6,24,25,29-32], depending on the design and operational mechanisms of the system. The response content of CAs was largely grounded in authoritative sources, including clinical guidelines, health websites, expert consensus, peer-reviewed literature, and evidence-based QnA libraries developed by study teams. Cloud-based platforms were also integrated in some studies to deliver responses from predefined knowledge bases, improving response consistency and system scalability [6,11,20,22,24,34]. Handover mechanisms were incorporated in only one-third of interventions, enabling escalation to clinical staff in high-risk situations to ensure safety and responsiveness [20,23,24,30,31,34]. Additionally, most interventions adopted mobile- or web-based interfaces, though only a third incorporated visual elements (eg, icons, avatars) [20,22,25,26,32].

This review supports the effectiveness of CAs in cancer care, particularly in promoting physical activity and alleviating symptoms, such as pain, treatment side effects, and anxiety, thereby enhancing patients’ overall quality of life. Although prior studies in chronic disease populations have also reported favorable outcomes with CAs, many of those reviews focused on relatively narrow objectives—such as physical activity or weight management—with limited attention to multidimensional outcomes [35,36]. In contrast, the cancer-specific CAs included in this review demonstrated broader functionality, addressing not only physical activity but also a wide range of symptoms and overall quality of life. This broader scope and effectiveness may be attributed to the interactive and tailored content of CAs, which aligns well with the complex and evolving needs of individuals undergoing cancer treatment [37]. Specifically, reductions in the symptom burden—such as pain, anxiety, and treatment-related side effects—highlight the potential of CAs to deliver symptom-specific education and practical self-care strategies [7]. Improvements in physical activity observed across studies may reflect the effectiveness of CA features, such as timely reminders, individualized feedback, and structured goal setting [38]. These benefits are particularly important in oncology, where preserving physical function and controlling symptoms are critical to maintaining quality of life [38]. Collectively, these features position CAs as a promising adjunct to conventional oncology care, with the potential to improve multidimensional health-related outcomes and enhance supportive care delivery.

However, the nonsignificant effect of CAs on depression and psychological distress requires further exploration. Both conditions represent complex, multifaceted, and long-lasting psychological challenges that require deeper emotional empathy and theoretically grounded interventions [39]. Although current CA systems may help alleviate transient psychological states, such as anxiety, by delivering multidimensional informational support, they remain inadequate for more complex and persistent psychological demands [37]. This inadequacy is largely attributable to the structural limitations of most existing CAs, which are predominantly rule based or minimally AI enhanced [10]. As a result, they are unable to recognize complex psychological states, respond empathetically, or support deep emotional regulation [40]. Notably, recent studies in other fields suggest that AI-powered CAs based on large language models (LLMs) may even outperform physicians in perceived empathy—possibly due to their advanced attention mechanisms, emotionally rich training data, and context-aware communication design [41]. Although these advances have not yet been applied in cancer care CAs, their integration holds promise. Future CA systems could incorporate emerging technologies, such as AI and LLMs, to enhance empathic communication and provide more comprehensive psychological support [10].

Narrative synthesis revealed that studies using single-session CA interventions primarily reported immediate informational benefits, specifically enhanced health information acquisition. In contrast, sustained health improvements—such as increased physical activity, better symptom management, and enhanced quality of life—were typically observed in studies that implemented multisession or extended-duration interventions lasting from 4 weeks to 6 months. These findings suggest that although brief, single-session interventions may suffice for improving informational outcomes, more sustained exposure—of at least 4 weeks—may be necessary to achieve meaningful improvements in sustained health benefits [8]. However, due to the limited number of studies per outcome and inconsistencies in intervention duration reporting, the optimal intervention duration could not be formally examined. Future research should investigate the relationship between intervention duration and effectiveness to determine the minimum exposure required for achieving sustained benefits across different domains of cancer care [42].

CAs were generally found to be safe, feasible, acceptable, and usable. However, suboptimal long-term engagement remains a critical challenge, underscoring the need for strategies to sustain continued use. This trend is consistent with findings from prior CA interventions in chronic disease management, where high attrition rates were commonly reported despite favorable early-phase implementation outcomes [43,44]. Several design features may have hindered this sustained engagement. Notably, two-thirds of included CAs were fully automated and lacked handover mechanisms to escalate complex or high-risk queries to clinical personnel, potentially weakening users’ sense of accountability and connection, thereby undermining long-term engagement [44]. In addition, only one-third incorporated visual elements (eg, avatars, icons), which may have limited interactivity and reduced the overall appeal of the interface [45]. Moreover, most CAs relied on narrow content databases, leading to repetitive information and diminishing novelty over time [10]. To address these issues, future CA interventions should embed health care professionals into the interaction loop, incorporate emotionally engaging visuals, and leverage advanced technologies—such as LLM-based interfaces and enriched data sources—to provide diverse, adaptive content that evolves with user needs [46]. These enhancements may improve long-term engagement, build trust, and strengthen the effectiveness and scalability of CA-based interventions [44].

### Strength and Limitations

This study presented the first systematic review and meta-analysis to comprehensively synthesize the characteristics, feasibility, and effectiveness of CA-based interventions in supporting care for patients with cancer, providing an up-to-date synthesis of the current evidence and inform future clinical practice and intervention development.

However, several limitations should be acknowledged. First, this review included only peer-reviewed studies published in English, which may have introduced publication and language bias (results are presented in Figures S9-S14 in Multimedia Appendix 1). This decision was made to ensure methodological rigor and consistency in reporting quality. However, relevant studies in other languages or nonindexed sources may have been overlooked. Future reviews could address this by incorporating non-English and gray literature to improve inclusiveness. Second, heterogeneity in intervention characteristics, such as intervention duration, technology algorithms, and delivery modality, may have introduced variability that warrants cautious interpretation. Given the limited number of eligible trials, formal subgroup analyses were not feasible; therefore, we interpreted outcome variability narratively based on key intervention features. Future meta-analyses with a larger evidence base may facilitate subgroup analyses to examine their potential moderating effects. Third, most of the included studies had relatively small sample sizes, and some were NRIs, which may limit the generalizability of the findings. This limitation reflects the early, exploratory stage of research on CA interventions in cancer care. To mitigate this, we conducted both meta-analysis and narrative synthesis to comprehensively integrate the current evidence on effectiveness. In addition, a separate narrative synthesis of implementation outcomes was performed to capture early insights into CA delivery and contextual applicability. Future well-designed RCTs with larger sample sizes are warranted to enhance the robustness of the evidence base and support broader clinical implementation.

### Future Direction

Overall, CA interventions address persistent service gaps in both clinical and community settings. However, their successful integration into routine care requires systematic consideration of diverse aspects. One key aspect identified in our review is the limited application of theoretical frameworks to guide the design, delivery, and evaluation of CA interventions. Future interventions should be guided by established theoretical frameworks to ensure coherent design, targeted content delivery, and meaningful outcome evaluation [47]. In particular, stage-based models, such as the Transtheoretical Model (TTM), which links intervention strategies and outcomes to users’ readiness for change (eg, precontemplation, preparation, action, maintenance), may offer added practical value [42]. Applying such models could help clarify how CAs influence patient outcomes, address individual needs, and reinforce the scientific foundation of future CA interventions [36].

Second, future CA development should build on existing technologies by incorporating more flexible, adaptive, and emotionally responsive capabilities. Many current systems remain limited by predefined scripts or limited AI functionality, restricting their ability to understand context or convey empathy. Integrating advanced techniques, such as LLMs, may improve natural language understanding and emotional responsiveness, leading to greater personalization and sustained user interaction [48]. Additionally, embedding health care professionals into the interaction loop and incorporating multimodal communication features may further enhance safety, accessibility, user trust, and long-term engagement [7].

Third, the successful application of general-purpose health CAs in oncology care highlights their potential for cross-condition adaptability and broader applicability across health care contexts. Although most CAs included in this review were specifically designed for oncology, several originally developed for general health conditions have also demonstrated effectiveness when adapted for oncology care [12,25,29]. This adaptability indicates that well-designed CAs may be transferable across clinical conditions, suggesting valuable directions for future development. Clinically, repurposing CAs may reduce development costs and expedite deployment in supportive care of different diseases [8]. This also supports the development of CA systems with reusable modules, where disease-specific content can be plugged into a standardized infrastructure [7]. This approach enables continuity of care across comorbid conditions by centralizing supportive functions in a unified CA platform. To support broader implementation, future research should examine how contextual adaptations (eg, population, disease, setting) affect CA effectiveness and user experience [49].

### Conclusion

This systematic review is the first to comprehensively evaluate CA interventions in supporting care for patients with cancer, with preliminary evidence supporting their feasibility and effectiveness. However, the limited psychological benefits and suboptimal long-term user engagement indicate the need for further refinement. Future research should adopt theory-driven approaches and explore the integration of emerging technologies to enhance personalization, empathy, and sustained engagement in CA interventions. Moreover, rigorous study designs—particularly large-scale RCTs—are needed to evaluate the effectiveness and implementation processes of CAs in cancer care.

## Appendix

Multimedia Appendix 1 Summary tables, figures, and supplementary information.

## Abbreviations

AI: artificial intelligence
CA: conversational agent
JBI: Joanna Briggs Institute
LLM: large language model
MD: mean difference
mHealth: mobile health
PRISMA: Preferred Reporting Items for Systematic Reviews and Meta-Analyses
QnA: question and answer
SMD: standardized mean difference
NRI: nonrandomized intervention
RCT: randomized controlled trial

## Data Availability

All data generated or analyzed during this study are included in this published paper and its supplementary information files.
