# Supplementary material for: The Effectiveness and Feasibility of Conversational Agents in Supporting Care for Patients With Cancer: Systematic Review and Meta-Analysis
Source: J Med Internet Res. 2025 Aug 8;27:e76968. doi: 10.2196/76968 (PMC12374140; doi:10.2196/76968)
Supplement: Multimedia Appendix 1 [file jmir_v27i1e76968_app1.docx]

**Multimedia Appendix**

**Table S1. PRISMA statement and checklist 2**

**Table S2. Search string 6**

**Figure S1. Comparison of fixed-effects and random-effects models in the meta-analysis of quality of life 8**

**Figure S2. Comparison of fixed-effects and random-effects models in meta-analysis of physical activity 8**

**Figure S3. Comparison of fixed-effects and random-effects models in meta-analysis of pain 9**

**Figure S4. Comparison of fixed-effects and random-effects models in meta-analysis of anxiety 9**

**Figure S5. Comparison of fixed-effects and random-effects models in meta-analysis of depression 10**

**Figure S6. Comparison of fixed-effects and random-effects models in meta-analysis of psychological distress 10**

**Figure S7. Methodological quality of randomized controlled trials 11**

**Figure S8. Risk of bias summary: randomized controlled trials 12**

**Table S3. Summary characteristics of included studies 13**

**Table S4. Feasibility, acceptability, usability of CA interventions 20**

**Table S5. Risk of bias in each quasi-experimental studies. 25**

**Figure S9. Funnel plot of publication bias for quality of life 26**

**Figure S10. Funnel plot of publication bias for physical activity 26**

**Figure S11. Funnel plot of publication bias for pain 27**

**Figure S12. Funnel plot of publication bias for anxiety 27**

**Figure S13. Funnel plot of publication bias for depression 28**

**Figure S14. Funnel plot of publication bias for psychological distress 28**

**Table S1 PRISMA statement and checklist**

| **Section and Topic** | **Item #** | **Checklist item** | **Location where item is reported** |
| --- | --- | --- | --- |
| **TITLE** | | |  |
| Title | 1 | Identify the report as a systematic review. | p 1 |
| **ABSTRACT** | | |  |
| Abstract | 2 | See the PRISMA 2020 for Abstracts checklist. | p 2-3 |
| **INTRODUCTION** | | |  |
| Rationale | 3 | Describe the rationale for the review in the context of existing knowledge. | p 3-5 |
| Objectives | 4 | Provide an explicit statement of the objective(s) or question(s) the review addresses. | p 5 |
| **METHODS** | | |  |
| Eligibility criteria | 5 | Specify the inclusion and exclusion criteria for the review and how studies were grouped for the syntheses. | p 6 |
| Information sources | 6 | Specify all databases, registers, websites, organisations, reference lists and other sources searched or consulted to identify studies. Specify the date when each source was last searched or consulted. | p 5-6 |
| Search strategy | 7 | Present the full search strategies for all databases, registers and websites, including any filters and limits used. | p 5-6 and Multimedia Appendix Table S2 |
| Selection process | 8 | Specify the methods used to decide whether a study met the inclusion criteria of the review, including how many reviewers screened each record and each report retrieved, whether they worked independently, and if applicable, details of automation tools used in the process. | p 6-7 |
| Data collection process | 9 | Specify the methods used to collect data from reports, including how many reviewers collected data from each report, whether they worked independently, any processes for obtaining or confirming data from study investigators, and if applicable, details of automation tools used in the process. | p 7 |
| Data items | 10a | List and define all outcomes for which data were sought. Specify whether all results that were compatible with each outcome domain in each study were sought (e.g. for all measures, time points, analyses), and if not, the methods used to decide which results to collect. | p 6-7 |
|  | 10b | List and define all other variables for which data were sought (e.g. participant and intervention characteristics, funding sources). Describe any assumptions made about any missing or unclear information. | p 7-8 |
| Study risk of bias assessment | 11 | Specify the methods used to assess risk of bias in the included studies, including details of the tool(s) used, how many reviewers assessed each study and whether they worked independently, and if applicable, details of automation tools used in the process. | p 7 |
| Effect measures | 12 | Specify for each outcome the effect measure(s) (e.g. risk ratio, mean difference) used in the synthesis or presentation of results. | p 7-8 |
| Synthesis methods | 13a | Describe the processes used to decide which studies were eligible for each synthesis (e.g. tabulating the study intervention characteristics and comparing against the planned groups for each synthesis (item #5)). | p 6 |
|  | 13b | Describe any methods required to prepare the data for presentation or synthesis, such as handling of missing summary statistics, or data conversions. | p 7-8 |
|  | 13c | Describe any methods used to tabulate or visually display results of individual studies and syntheses. | p 7-8 |
|  | 13d | Describe any methods used to synthesize results and provide a rationale for the choice(s). If meta-analysis was performed, describe the model(s), method(s) to identify the presence and extent of statistical heterogeneity, and software package(s) used. | p 7-8 |
|  | 13e | Describe any methods used to explore possible causes of heterogeneity among study results (e.g. subgroup analysis, meta-regression). | - |
|  | 13f | Describe any sensitivity analyses conducted to assess robustness of the synthesized results. | P 8 |
| Reporting bias assessment | 14 | Describe any methods used to assess risk of bias due to missing results in a synthesis (arising from reporting biases). | p 8 |
| Certainty assessment | 15 | Describe any methods used to assess certainty (or confidence) in the body of evidence for an outcome. | - |
| **RESULTS** | | |  |
| Study selection | 16a | Describe the results of the search and selection process, from the number of records identified in the search to the number of studies included in the review, ideally using a flow diagram. | p 7-8 |
|  | 16b | Cite studies that might appear to meet the inclusion criteria, but which were excluded, and explain why they were excluded. | p 8-9 |
| Study characteristics | 17 | Cite each included study and present its characteristics. | p 10-11 |
| Risk of bias in studies | 18 | Present assessments of risk of bias for each included study. | p 19-20 |
| Results of individual studies | 19 | For all outcomes, present, for each study: (a) summary statistics for each group (where appropriate) and (b) an effect estimate and its precision (e.g. confidence/credible interval), ideally using structured tables or plots. | p 15-18 |
| Results of syntheses | 20a | For each synthesis, briefly summarise the characteristics and risk of bias among contributing studies. | p 15-17 |
|  | 20b | Present results of all statistical syntheses conducted. If meta-analysis was done, present for each the summary estimate and its precision (e.g. confidence/credible interval) and measures of statistical heterogeneity. If comparing groups, describe the direction of the effect. | p 15-17 |
|  | 20c | Present results of all investigations of possible causes of heterogeneity among study results. | - |
|  | 20d | Present results of all sensitivity analyses conducted to assess the robustness of the synthesized results. | p15 |
| Reporting biases | 21 | Present assessments of risk of bias due to missing results (arising from reporting biases) for each synthesis assessed. | p 15 |
| Certainty of evidence | 22 | Present assessments of certainty (or confidence) in the body of evidence for each outcome assessed. | - |
| **DISCUSSION** | | |  |
| Discussion | 23a | Provide a general interpretation of the results in the context of other evidence. | p 20 |
|  | 23b | Discuss any limitations of the evidence included in the review. | p 23-24 |
|  | 23c | Discuss any limitations of the review processes used. | p 23-24 |
|  | 23d | Discuss implications of the results for practice, policy, and future research. | p 24-25 |
| **OTHER INFORMATION** | | |  |
| Registration and protocol | 24a | Provide registration information for the review, including register name and registration number, or state that the review was not registered. | p 3 |
|  | 24b | Indicate where the review protocol can be accessed, or state that a protocol was not prepared. | p 5 |
|  | 24c | Describe and explain any amendments to information provided at registration or in the protocol. | - |
| Support | 25 | Describe sources of financial or non-financial support for the review, and the role of the funders or sponsors in the review. | p 25 |
| Competing interests | 26 | Declare any competing interests of review authors. | p 25 |
| Availability of data, code and other materials | 27 | Report which of the following are publicly available and where they can be found template data collection forms; data extracted from included studies; data used for all analyses; analytic code; any other materials used in the review. | - |

**Table S2 Search string**

| **Database** | **Number of articles retrieved** |
| --- | --- |
| **Web of Science** | 499 |
| 1. TS= ("Generative Artificial Intelligence" OR “digital assistant*” OR “digital agent*” OR “ai bot*” OR “virtual coach” OR “artificial agent*” OR “virtual agent*” OR “virtual assistant*” OR “assistance technolog*” OR “social robot*” OR “conversational agency” OR “conversational AI” OR “nurse avatar*” OR “nursing avatar*” OR “virtual avatar*” OR chatterbot* OR “social bot*” OR “chat robot*” OR “chat bot*” OR chatbot* OR “relational agent*” OR “dialogue system*” OR “dialog system*” OR “conversational system*” OR “conversational agent*” OR “Question answer* system*”) 2. TS= (cancer* OR neoplasm* OR carcinoma* OR oncol* or malignan* OR tumor* OR leukemia* OR leukaemia* or sarcoma* OR lymphoma* OR melanoma* OR blastoma* OR myeloma*) 3. #1 AND #2 |  |
| **PubMed** | 294 |
| 1. "Generative Artificial Intelligence"[Mesh] OR digital assistant*[Title/Abstract] OR digital agent*[Title/Abstract] OR ai bot*[Title/Abstract] OR virtual coach[Title/Abstract] OR artificial agent*[Title/Abstract] OR virtual agent*[Title/Abstract] OR virtual assistant*[Title/Abstract] OR assistance technolog*[Title/Abstract] OR social robot*[Title/Abstract] OR conversational agency[Title/Abstract] OR conversational AI[Title/Abstract] OR nurse avatar*[Title/Abstract] OR nursing avatar*[Title/Abstract] OR virtual avatar*[Title/Abstract] OR chatterbot*[Title/Abstract] OR social bot*[Title/Abstract] OR chat robot*[Title/Abstract] OR chat bot*[Title/Abstract] OR chatbot*[Title/Abstract] OR relational agent*[Title/Abstract] OR dialogue system*[Title/Abstract] OR dialog system*[Title/Abstract] OR conversational system*[Title/Abstract] OR conversational agent*[Title/Abstract] OR Question answer* system*[Title/Abstract] 2. ("Neoplasms"[Mesh]) OR (cancer*[Title/Abstract] OR neoplasm*[Title/Abstract] OR carcinoma*[Title/Abstract] OR oncol*or malignan*[Title/Abstract] OR tumor*[Title/Abstract] OR leukemia*[Title/Abstract] OR leukaemia*or sarcoma*[Title/Abstract] OR lymphoma*[Title/Abstract] OR melanoma*[Title/Abstract] OR blastoma*[Title/Abstract] OR myeloma*[Title/Abstract]) 3. #1 AND #2 |  |
| **Embase** | 470 |
| 1. ('malignant neoplasm' OR cancer* OR 'neoplasm' OR neoplasm* OR 'carcinoma' OR carcinoma* OR oncol* OR malignan* OR tumor* OR 'leukemia' OR leukemia* OR leukaemia* OR 'sarcoma' OR sarcoma* OR 'lymphoma' OR lymphoma* OR 'melanoma' OR melanoma* OR 'blastoma' OR blastoma* OR 'myeloma' OR myeloma*):ab,ti 2. ('generative artificial intelligence' OR 'digital assistant*' OR 'digital agent*' OR 'ai bot*' OR 'virtual coach' OR 'artificial agent*' OR 'virtual agent*' OR 'virtual assistant*' OR 'assistance technolog*' OR 'social robot' OR 'social robot*' OR 'conversational agency' OR 'conversational ai' OR 'nurse avatar*' OR 'nursing avatar*' OR 'virtual avatar*' OR chatterbot* OR 'social bot*' OR 'chat robot*' OR 'chat bot*' OR 'chatbot' OR chatbot* OR 'relational agent*' OR 'dialogue system*' OR 'dialog system*' OR 'conversational system*' OR 'conversational agent*' OR 'question answer* system*'):ab,ti 3. #1 AND #2 |  |
| **Cochrane library** | **824** |
| 1. MeSH descriptor: [Neoplasms] explode all trees 2. ((cancer* OR neoplasm* OR carcinoma* OR oncol* or malignan* OR tumor* OR leukemia* OR leukaemia* or sarcoma* OR lymphoma* OR melanoma* OR blastoma* OR myeloma*)):ti,ab,kw 3. MeSH descriptor: [Artificial Intelligence] explode all trees 4. (("Generative Artificial Intelligence" OR “digital assistant*” OR “digital agent*” OR “ai bot*” OR “virtual coach” OR “artificial agent*” OR “virtual agent*” OR “virtual assistant*” OR “assistance technolog*” OR “social robot*” OR “conversational agency” OR “conversational AI” OR “nurse avatar*” OR “nursing avatar*” OR “virtual avatar*” OR chatterbot* OR “social bot*” OR “chat robot*” OR “chat bot*” OR chatbot* OR “relational agent*” OR “dialogue system*” OR “dialog system*” OR “conversational system*” OR “conversational agent*” OR “Question answer* system*”)): ti,ab,kw 5. (#1 or #2) AND (#4 or #5) |  |


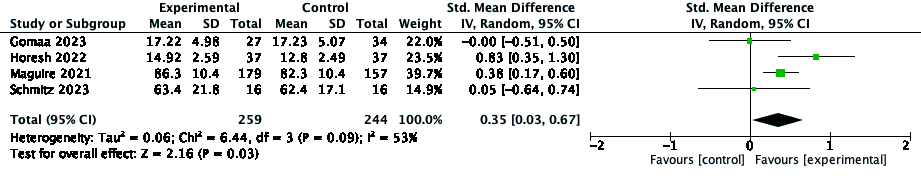

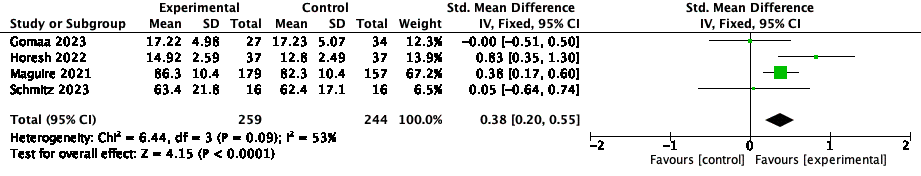


**Figure S1. Comparison of fixed-effects and random-effects models in meta-analysis of quality of life**


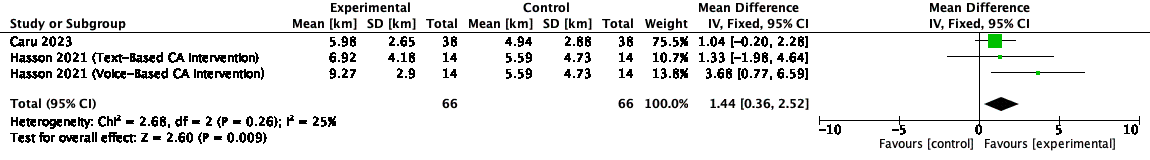

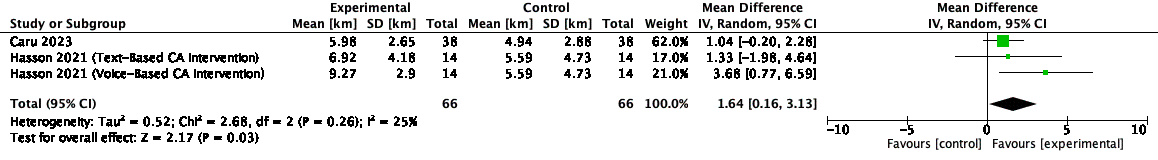


**Figure S2. Comparison of fixed-effects and random-effects models in meta-analysis of physical activity**


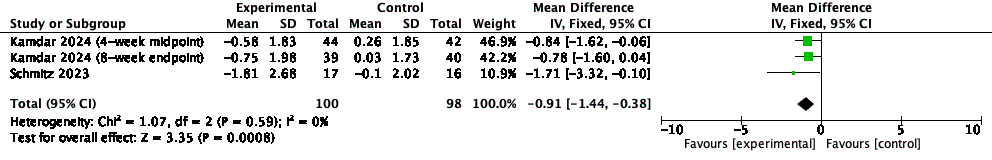

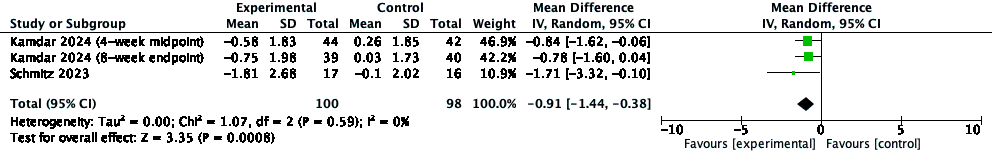


**Figure S3. Comparison of fixed-effects and random-effects models in meta-analysis of pain**


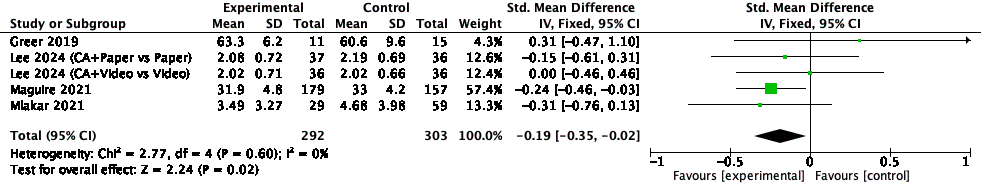

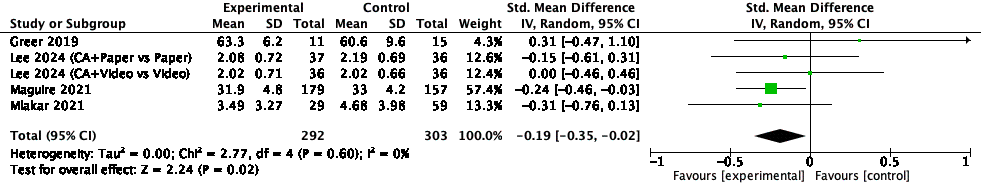


**Figure S4. Comparison of fixed-effects and random-effects models in meta-analysis of anxiety**


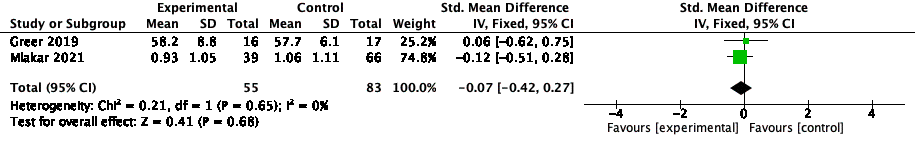

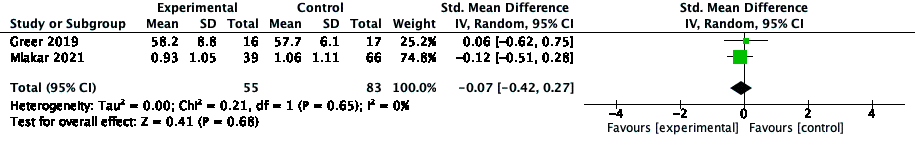


**Figure S5. Comparison of fixed-effects and random-effects models in meta-analysis of depression**


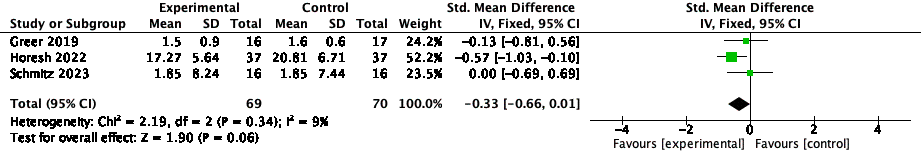

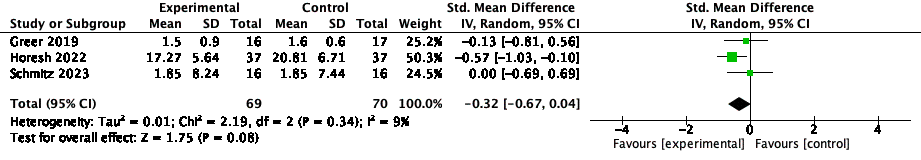


**Figure S6. Comparison of fixed-effects and random-effects models in meta-analysis of psychological distress**

**
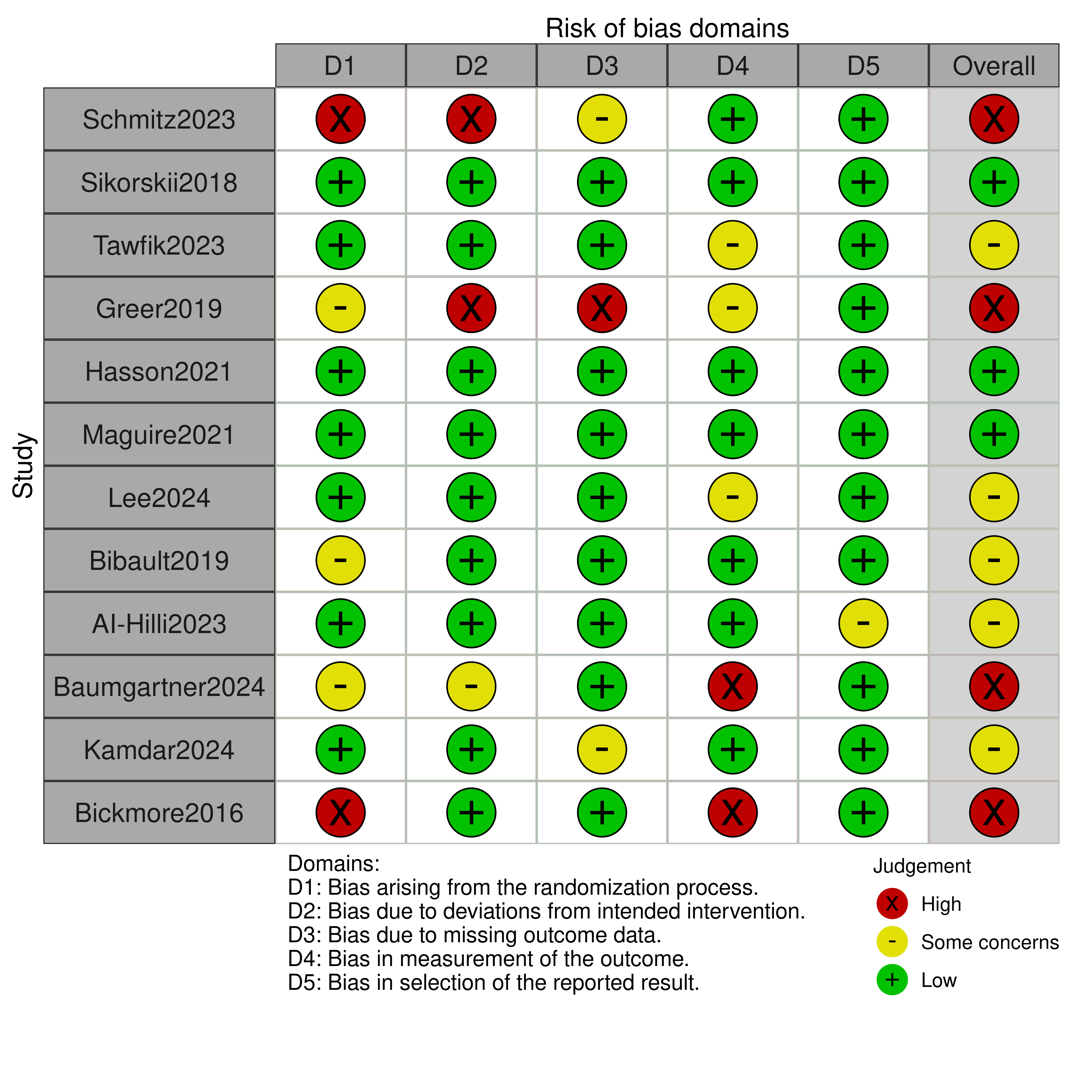
**

**Figure S7. Methodological quality of randomized controlled trials**


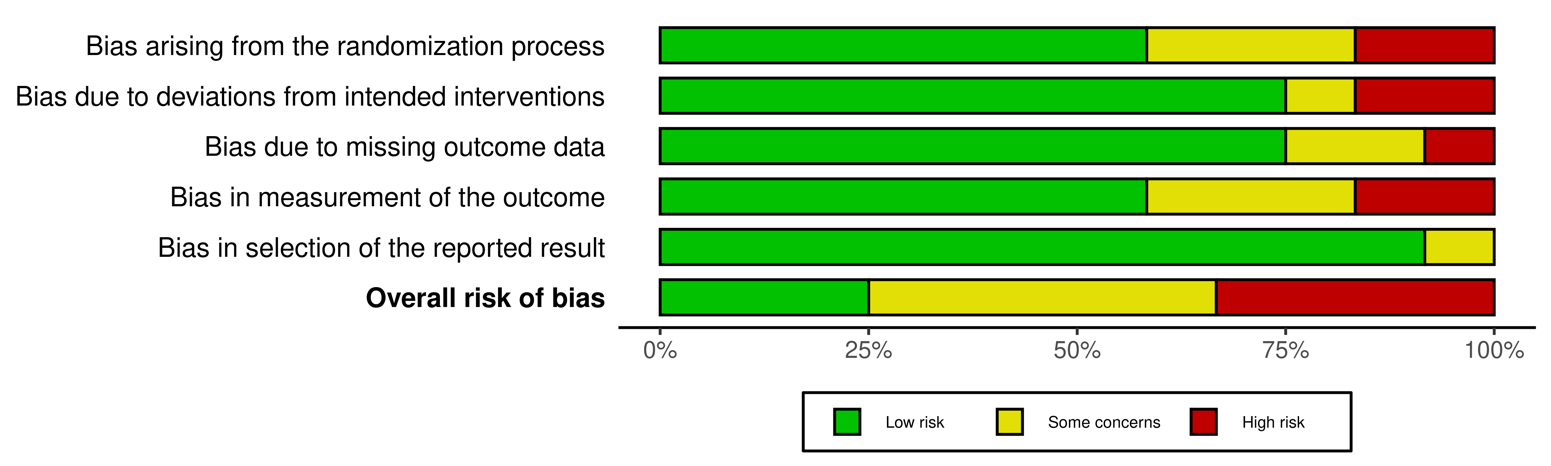


**Figure S8. Risk of bias summary: randomized controlled trials**

**Table S3. Summary characteristics of included studies**

| **Study** | **Intervention components** | | | |  | | **CA delivery and interaction logic** | | | | | | **Input and output information** | | | |
| --- | --- | --- | --- | --- | --- | --- | --- | --- | --- | --- | --- | --- | --- | --- | --- | --- |
|  | **Consultable content** | **CA-only vs. multi-component** | **Duration/**  **frequency** | **Theory-Guided** | **CA name** | **Delivery Platform** | | **Prompt sequence** | **Cancer**  **specific CA** | **Type of embodiment** | **Algorithm type** | **Clinician Referral** | **Input Data** | **Input Format** | **Input/**  **output Modality** | **Output Source** |
| Schmitz et al [1] | General breast cancer management knowledge | Multicomponent CA-based intervention (medical team referral for severe symptoms） | 6 months/  daily | None | Nurse AMIE | Amazon Echo Show and Alexa | | Hybrid (both System- and user-initiated) | Yes | Embodied | AI-based | Yes | Symptoms and physical activity | Mixed input (including button and free input) | Speech | AICR^4^ website, Amazon Alexa API |
| Sikorskii et al [2] | Symptom management and medication reminder | CA-only intervention | 8 weeks/ daily or each day | None | IVR | Phone calls | | System-initiated | Yes | Disembodied | Rule-based | No | Adherence to Oral Oncolytic Agents, symptoms | Button input | Speech | NCCN guideline^3^ |
| Tawfik et al [3] | Symptom management | CA-only intervention | 4 months | Empowerment education | ChemoFreeBot | Microsoft Azure | | User-initiated | Yes | Disembodied | Rule-based | No | Adverse effects of chemotherapy | Free input | Text | Study-developed content library based on FAQs^8^ and ACS guidelines^5^, Microsoft QnA Maker API |
| Greer et al [4] | Psychology management | CA-only intervention | 4 weeks | Stress and coping theory and broaden-and-build theory | Vivibot | Facebook Messenger | | System-initiated | No | Disembodied | Rule-based | No | Not specified | Mixed input | Multimodal (text, speech, and vision) | Eight positive psychological skills from literature |
| Hassoon et al [5] | Physical activity promotion | Multicomponent CA-based intervention (CA combined with wearable sensor) | 4 weeks | None | Int1: MyCoach; Int 2: Coachtext | Int1: Amazon Echo/Alexa smart speaker Int 2: Short Message Service (SMS) | | Int1: User-initiated Int 2: Hybrid | Yes | Int 1: Embodied Int 2: Disembodied | Int 1: AI-based Int2: AI-based | No | Physical activity | Int 1: Free input Int 2: Button input | Int 1: Multimodal  Int 2: text | Study-developed content library based on literature, Amazon Alexa API |
| Maguire et al [6] | Symptom management | Multicomponent CA-based intervention (medical team referral for severe symptoms） | 6 cycles of chemother-apy | None | ASyMS | A mHealth App (Connectivity Logger app) | | Hybrid | Yes | Disembodied | Rule-based | Yes | Adjuvant chemotherapy related side effects | Button input | Multimodal | Local, national, and European best practice guidelines |
| Lee et al [7] | Education regarding breast cancer radiotherapy | Multicomponent CA-based intervention (combined with other education materials such as texts or videos） | Throughout radiation therapy | None | None | Kakao | | User-initiated | Yes | Disembodied | Hybrid (includes both AI-based and rule-based algorithm) | Yes | Radiation procedure questions, side effects, and lifestyle behaviors | Mixed input | Multimodal | Study-developed content library based on interview-informed QnA^9^, Kakao chatbot API |
| Horesh et al [8] | Psychology management | Multicomponent CA-based intervention (combined with mindfulness therapy and virtual reality) | 1 month | Cognitive Behavioral Therapy | Luna | A mHealth App (Bubble) | | Hybrid | No | Embodied | Hybrid | No | Psychotherapy feedback and planning | Button input | Text | Study-developed content library based on literature and Korean Breast Cancer Society guidelines |
| Mlakar et al [9,10] | General breast cancer management knowledge | Multicomponent CA-based intervention (mHealth app and a smart band) | 6 months/  daily | None | Eva | A mHealth App (PERSIST) | | System -initiated | Yes | Embodied | AI-based | No | Not specified | Mixed input | Multimodal | NCCN guidelines^3^ |
| Kamdar et al [11] | Pain management | Multicomponent CA-based intervention (CA combined with prescription refill request tool etc.） | 8 weeks | None | ePAL | a mHealth App (ePAL) | | Hybrid | Yes | Disembodied | Hybrid | Yes | Pain-related questions | Mixed input | Text | Study-developed content library based on literatures |
| Al-Hilli et al [12] | Genetic counseling education | CA-only intervention | 1 session | None | Gia | SMS | | Hybrid | No | Disembodied | Hybrid | No | Pre-testing genetic counseling | Mixed input | Text | Expert opinion of a genetics counselor |
| Baumgärtner et al [13] | General prostate cancer management knowledge | CA-only intervention | 4 weeks | None | PROSCA | Website based on SAP Conversational AI (SAP CAI) | | Hybrid | Yes | Disembodied | Hybrid | No | Prostate diseases, diagnostic procedures, treatment options, and symptom checking | Mixed input | Text | EAU guidelines^7^, high-quality literatures, and SAP CAI |
| Bibault et al [14] | General breast cancer management knowledge | CA-only intervention | 1 session | None | Vik | Facebook Messenger | | User-initiated | Yes | Disembodied | AI-based | No | Nutrition, sport, symptoms, treatments or appointment | Free input | Text | Literature-based knowledge |
| Queiroz et al [15] | General colorectal cancer management knowledge | Multicomponent CA-based intervention (CA combined with wearable device) | 8 weeks | None | Dialog  flow | Facebook Messenger | | Hybrid | Yes | Disembodied | Hybrid | Yes | Symptoms， adverse effects, physical activity, food, and questionnaires | Mixed input | Text | ACS guidelines^5^ |
| Bickmore et al [16] | Clinical trial information | CA-only intervention | 1 session | None | None | A Tablet app | | System-initiated | Yes | Embodied | Hybrid | No | Clinical trial information | Button input | Multimodal | NCI resources^6^ |
| Gomaa et al [17] | Symptom management | CA-only intervention | 2 months/ 3 times per week | None | None | SMS | | Hybrid | Yes | Disembodied | Rule-based | No | Symptoms, emotion, pre-treatment and during treatment information | Button input | Text | Literature review, expert consensus, NCI guidelines^6^ |
| Caru et al [18] | General breast cancer management knowledge | Multicomponent CA-based intervention (CA combined with mindfulness therapy etc.） | 90 days/daily | None | Nurse AMIE | Amazon Echo Show and Alexa | | Hybrid | Yes | Embodied | AI-based | Yes | Symptoms (sleep problem, distress, fatigue, pain) and physical activity | Mixed input | Speech | AICR4 website, Amazon Alexa API |

^1^ CA: conversational agent

^2^ Int: intervention

^3^ NCCN: National Comprehensive Cancer Network

^4^ AICR: American Institute for Cancer Research

^5^ ACS: American cancer society

^6^ NCI: National Cancer Institute

^7^ EAU: European Association of Urology

^8^ FAQs: Frequently Asked Questions

^9^ QnA: question and answer

**Table S4. Feasibility, acceptability, usability of conversational agent interventions**

| **Study** | **Feasibility** | **Acceptability** | **Usability** |
| --- | --- | --- | --- |
| Schmitz et al [1] | (1) Retention rate:  Results: 85.7%  (2) Safety  Results: No adverse events were reported by any participants in this study. | (1). Satisfaction:  a) Client Satisfaction Questionnaire, range: 8-32 Results: the mean score was 25.36 b) Rate of participants who reported satisfaction with the CA Results: 70% or higher. (2). Recommendation: (The Credibility/Expectancy Questionnaire, range: 0-10) Results: the mean score was 7.24. (3). Perceived Helpfulness: (The Credibility/Expectancy Questionnaire, range: 0-10) Results: participants answered a mean of 5.76. | (1) Usability of content: (User Version of the Mobile Application Rating Scale, range: 0 to 100) Results: the mean score was 86.14 |
| Tawfik et al [3] | NA | NA | (1) Usability of content  Results:  (a) 94% participants responses were useful, appropriate and informative (2) Easy to use:  Results: 94% agreed that the CA is easy to use (3) User experience:  Results:  (a) 72% found that the CA’s personality was realistic and engaging  (b) 76% found that the CA coped well with any errors or mistakes they made |
| Greer et al [4] | (1) Retention rate:  Results: 64.0%  (2) Interactions Results: Average interactions: 12.1 (SD 7.1) sessions (3) Duration of engagement  Results: Average duration of conversation: 73.8 (SD 52) minutes | (1) Perceived Helpfulness:  Results: Single-item helpfulness assessment (range: 0-3), average score: 2.03 (SD 0.72). (2) Recommendation Results: Single-item recommendation assessment (range: 0-10), average rating of 6.9 (SD 2.6). | (1) User experience  Results: Participants appreciated the CA's nonjudgmental nature, the ability to share experiences, and the positive psychology content. |
| Hassoon et al [5] | (1) Safety  Results: There were no adverse events during the intervention period. (2) Interactions Results: (a) Intervention 1 (MyCoach): participants had an average of 2 interactions per day.  (b) Intervention 2 (SmartText): Participants received 3 text messages per day, except one participant who did not get messages on 1 day. | NA | NA |
| Maguire et al [6] | (1) Retention rate:  Results: 69.4%  (2) Safety  Results: No adverse events were reported by any participants in this study. | NA | NA |
| Lee et al [7] | (1) Interactions Results: (a) 446 interactions completed, average interactions per user: 6.1 (b) Chat inquiries were about specific symptoms, like persistent thirst, throat pain, and the effectiveness of oriental medicine for immunity | NA | NA |
| Horesh et al [8] |  | (1) Satisfaction  Results:  (a) 97% of participants reported enjoying the intervention experience  (b) 94% were satisfied with the home-based use of the intervention  (2) Perceived Helpfulness: Results: 70% indicated that the intervention helped them return to their daily routine  (3) Recommendation  Results: 97% |  |
| Al-Hilli et al [12] | NA | (1) Satisfaction (self-designed questionnaire, range: 6–30) Results: The median satisfaction score of CA was 30, showing no significant difference compared to in-person consultations. | NA |
| Baumgärtner et al [13] | (1) Retention rate:  Results: 72.9%  (2) Interactions Results:  (a) the CA facilitated 176 conversations, average interactions per user: 3.0 (b) on average, each user generated 7.1 inputs per conversation. (3) Duration of engagement  Results: The average duration of CA use per person = 3.4 d (maximum: 14d)  (4) Engagement rate  Results: 79.6% participants stated that they used PROSCA at least once. | (1) Perceived Helpfulness: Results:  (a) 71.4-73.2% fully to partially agree that they gained substantial information (b) 39 (90.7%) would like to use the CA again during another hospital visit (2) Recommendation Results: 41 (95.3%) recommend the general application of the CA in clinical routine. | (1) Easy to use: Results:  (a) 36 (83.7%) stated not needing any help when using the CA, 5 (11.6%) agreed partly, with 2 (4.7%) requiring ongoing technically help.  (b) All users rated the CA as easy to use. |
| Kamdar et al [11] | (1) Retention rate:  Results: 71.4% (2) Interactions Results: (a) patients utilized the CA on average 2.1 times per week. (b) The average usage frequency dropped from 3.5 to 1.5 days per week by week 8. (3) Engagement rate Results:  (a) weekly engagement dropping from 86% to 48% by week 8. (b) 14.3% of patients randomized to the CA group did not engage at all with the app.  (c) 47.6% of participants interacted with the CA at least once per week. | NA | (1) Easy to use:  Results:  (a) a mean score of 8.6 (SD = 1.80) on ease of understanding (assessed by an item ranged 0-10),  (b) a mean score of 8.63 (SD = 1.66) on ease of use (assessed by an item ranged 0-10),  (2) Usability of content (assessed by an item ranged 0-10) Results: a mean score of 7.8 (SD = 2.4) on the overall usefulness. |
| Queiroz et al [15] | (1) Retention rate:  Results: 84.6%  (2) Engagement rate Results: (a) 69.2% patients used at least 4 out of 8 weeks. (b) 61.5% patients used at least 5 weeks. | NA | (1) User experience (User Experience Questionnaire, range -3 to 3) Results: Most scores exceeded the benchmark. Attractiveness (1.95) and efficiency (2.14) were rated excellent. Perspicuity (1.96), dependability (1.64), and stimulation (1.68) were rated good. Novelty (0.43) was below average, with 50% of benchmark results scoring higher. (2) Usability of content (System Usability Scale, 0-100) Results: The mean score was 79.6 ± 8.8, above the 68-point threshold for acceptable usability |
| Bickmore et al [16] | NA | (1). Satisfaction:  Results: (Scale self-report item, range 1-7, 7 is the best) (a) Participants were significantly more satisfied with the CA than the conventional interface (mean 4.9 ± 1.8 vs. 3.2 ± 1.8, U=363, P<.001). (b) They reported feeling less frustrated (mean 2.1 ± 1.7 vs. 3.7 ± 2.2, U=405, P<.001). (c) They also felt more pleased after completing the task (mean 5.1 ± 2.1 vs. 3.4 ± 1.9, U=380, P<.001). | NA |
| Gomaa et al [17] | (1) Retention rate:  Results: 79.4% (2) Interactions Results: Over 2 months, Message exchanges ranged from 1 to 33, with an average of 15 (SD 4) per patient.  (3) Engagement rate Results: Over 2 months, 59% (n = 20) of patients actively engaged, texting back at least once. | (1) Perceived Helpfulness: (a self-reported questionnaire ranged: 1-5 points)  Results: a mean score of 3.73 (SD 0.85) | (1) Usability of content: (a self-reported questionnaire ranged 1-5 points) Results: a mean score of 4.47 (SD 0.45) (2) User experience:  (a) Nonjudgmental nature.  (b) Effective error management (c) Coping with side effects, fostering empowerment. (d) The encouraging messages provided solace (e) Easy to use, enhancing decision-making. (f) Improve patients’ communication skills in interactions with medical staff (g) The personalized approach fitted individual needs |
| ^1^CA: conversational agent | | | |

| Study (First Author, year) | Q1 | Q2 | Q3 | Q4 | Q5 | Q6 | Q7 | Q8 | Q9 | Total |
| --- | --- | --- | --- | --- | --- | --- | --- | --- | --- | --- |
| Horesh et al, 2022 [8] | YES | YES | YES | NO | NO | YES | YES | YES | YES | 7 |
| Mlakar et al, 2021 [9,10] | YES | YES | YES | NO | YES | NO | YES | YES | YES | 7 |
| Caru et al, 2023 [18] | YES | YES | YES | NO | YES | YES | YES | YES | YES | 8 |
| Queiroz et al, 2023 [15] | YES | YES | UNCLEAR | YES | YES | YES | YES | YES | YES | 8 |
| Gomaa et al, 2023 [17] | YES | YES | YES | NO | NO | YES | YES | YES | YES | 7 |
| **Note:**  ^a^Q1. Is it clear in the study what is the ‘cause’ and what is the ‘effect’ (i.e. there is no confusion about which variable comes first)?  ^b^Q2.Were the participants included in any comparisons similar?  ^c^Q3.Were the participants included in any comparisons receiving similar treatment/care, other than the exposure or intervention of interest?  ^d^Q4.Was there a control group?  ^e^Q5.Were there multiple measurements of the outcome both pre and post the intervention/exposure?  ^f^Q6. Was follow up complete and if not, were differences between groups in terms of their follow up adequately described and analyzed?  ^g^Q7. Were the outcomes of participants included in any comparisons measured in the same way?  ^h^Q8. Were outcomes measured in a reliable way?  ^i^Q9. Was appropriate statistical analysis used? | | | | | | | | | | |

**Table S5. Risk of Bias in each quasi-experimental studies.**

**
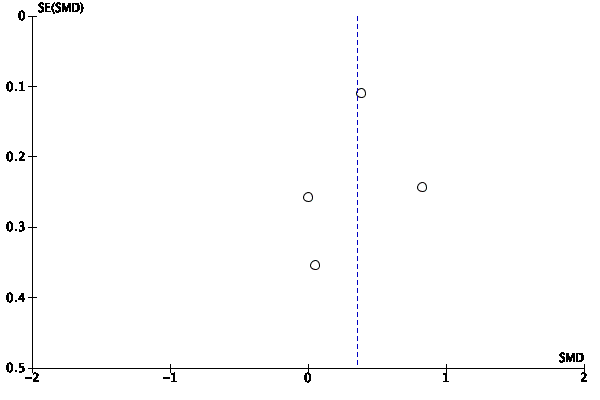
**

**Figure S9. Funnel plot of publication bias for quality of life**

**
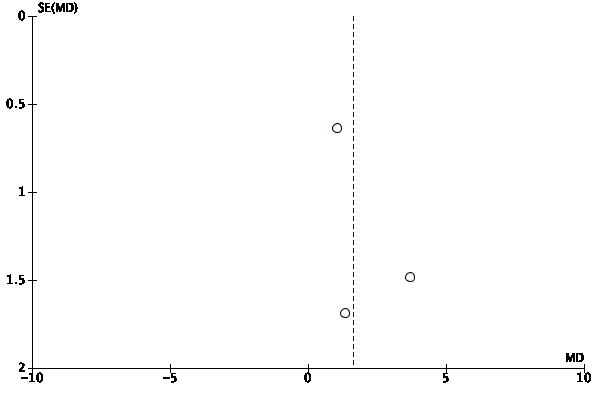
**

**Figure S10. Funnel plot of publication bias for physical activity.**

**
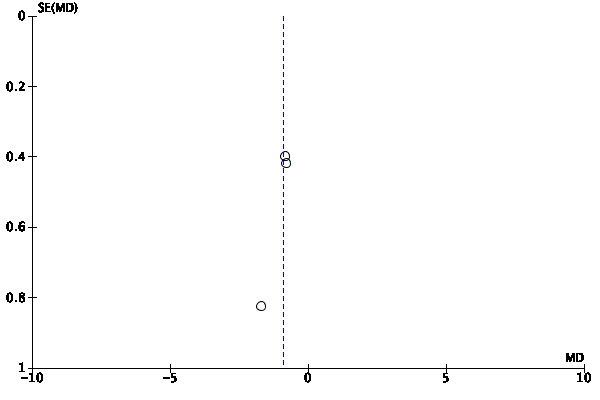
**

**Figure S11. Funnel plot of publication bias for pain scores.**

**
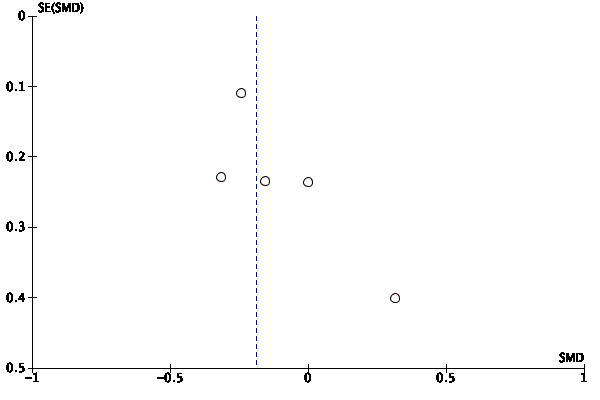
**

**Figure S12. Funnel plot of publication bias for anxiety.**

**
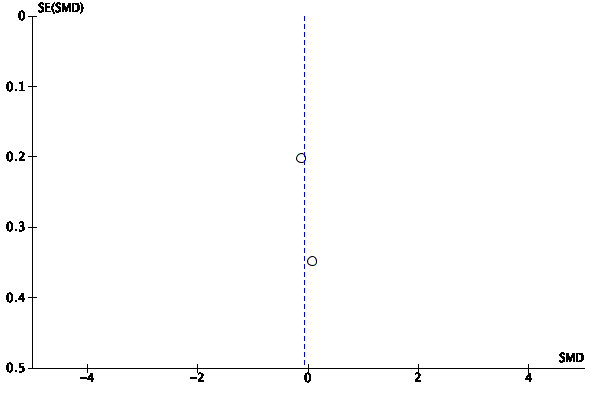
**

**Figure S13. Funnel plot of publication bias for depression.**

**
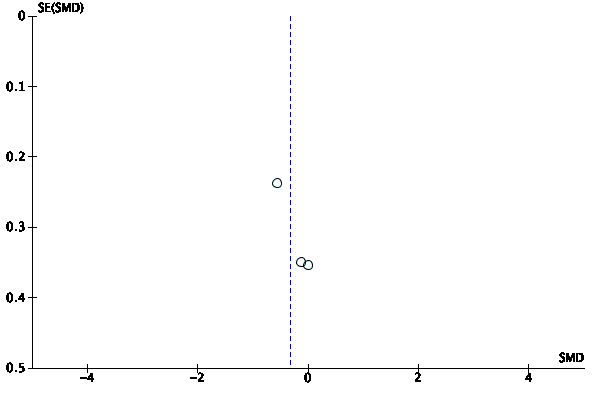
**

**Figure S14. Funnel plot of publication bias for psychological distress.**

## References

1. Schmitz KH, Kanski B, Gordon B, Caru M, Vasakar M, Truica CI, et al. Technology-based supportive care for metastatic breast cancer patients. *Support Care Cancer*. 2023 Jun 20;31(7):401. PMID: 37338627. doi: 10.1007/s00520-023-07884-3.
2. Sikorskii A, Given CW, Given BA, Vachon E, Krauss JC, Rosenzweig M, et al. An automated intervention did not improve adherence to oral oncolytic agents while managing symptoms: results from a two-arm randomized controlled trial. J Pain *Symptom Manag*. 2018 Nov;56(5):727-35. PMID: 30096441. doi: 10.1016/j.jpainsymman.2018.07.021.
3. Tawfik E, Ghallab E, Moustafa A. A nurse versus a chatbot ‒ the effect of an empowerment program on chemotherapy-related side effects and the self-care behaviors of women living with breast cancer: a randomized controlled trial. *BMC Nurs*. 2023 Apr 6;22(1):102. PMID: 37024875. doi: 10.1186/s12912-023-01243-7.
4. Greer S, Ramo D, Chang YJ, Fu M, Moskowitz J, Haritatos J. Use of the chatbot "Vivibot" to deliver positive psychology skills and promote well-being among young people after cancer treatment: randomized controlled feasibility trial. *JMIR Mhealth Uhealth*. 2019 Oct 31;7(10):e15018. PMID: 31674920. doi: 10.2196/15018.
5. Hassoon A, Baig Y, Naiman DQ, Celentano DD, Lansey D, Stearns V, et al. Randomized trial of two artificial intelligence coaching interventions to increase physical activity in cancer survivors. *npj Digit Med*. 2021 2021/12/09;4(1):168. doi: 10.1038/s41746-021-00539-9.
6. Maguire R, McCann L, Kotronoulas G, Kearney N, Ream E, Armes J, et al. Real time remote symptom monitoring during chemotherapy for cancer: European multicentre randomised controlled trial (eSMART). *BMJ*. 2021 Jul 21;374:n1647. PMID: 34289996. doi: 10.1136/bmj.n1647.
7. Lee J, Byun HK, Kim YT, Shin J, Kim YB. A study on breast cancer patient care using chatbot and video education for radiation therapy: a randomized controlled trial. Int *J Radiat Oncol Biol Phys*. 2024. doi: 10.1016/j.ijrobp.2024.12.012.
8. Horesh D, Kohavi S, Shilony-Nalaboff L, Rudich N, Greenman D, Feuerstein JS, et al. Virtual reality combined with artificial intelligence (VR-AI) reduces hot flashes and improves psychological well-being in women with breast and ovarian cancer: a pilot study. *Healthcare*. 2022;10(11):2261. PMID: doi:10.3390/healthcare10112261.
9. Mlakar I, Lin S, Aleksandraviča I, Arcimoviča K, Eglītis J, Leja M, et al. Patients-centered SurvivorShIp care plan after cancer treatments based on big data and artificial intelligence technologies (PERSIST): a multicenter study protocol to evaluate efficacy of digital tools supporting cancer survivors. *BMC Med Inform Decis Mak*. 2021 Aug 14;21(1):243. PMID: 34391413. doi: 10.1186/s12911-021-01603-w.
10. Bema, D. Full clinical study validation results of PERSIST. 2023. Zenodo. Accessed July 31, 2025 https://zenodo.org/records/8232106
11. Kamdar M, Jethwani K, Centi AJ, Agboola S, Fischer N, Traeger L, et al. A digital therapeutic application (ePAL) to manage pain in patients with advanced cancer: a randomized controlled trial. *J Pain Symptom Manag*. 2024 Sep;68(3):261-71. PMID: 38866116. doi: 10.1016/j.jpainsymman.2024.05.033.
12. Al-Hilli Z, Noss R, Dickard J, Wei W, Chichura A, Wu V, et al. A randomized trial comparing the effectiveness of pre-test genetic counseling using an artificial intelligence automated chatbot and traditional in-person genetic counseling in women newly diagnosed with breast cancer. *Ann Surg Oncol*. 2023 Oct;30(10):5990-6. PMID: 37567976. doi: 10.1245/s10434-023-13888-4.
13. Baumgärtner K, Byczkowski M, Schmid T, Muschko M, Woessner P, Gerlach A, et al. Effectiveness of the medical chatbot PROSCA to inform patients about prostate cancer: results of a randomized controlled trial. *Eur Urol Open Sci*. 2024 Nov;69:80-8. PMID: 39329071. doi: 10.1016/j.euros.2024.08.022.
14. Bibault JE, Chaix B, Guillemassé A, Cousin S, Escande A, Perrin M, et al. A Chatbot versus physicians to provide information for patients with breast cancer: blind, randomized controlled noninferiority trial. *J Med Internet Res*. 2019 Nov 27;21(11):e15787. PMID: 31774408. doi: 10.2196/15787.
15. Queiroz D, Passarello R, Fé V, Rossini A, Silveira E, Queiroz E, et al. A wearable chatbot-based model for monitoring colorectal cancer patients in the active phase of treatment. *Healthc Analytics*. 2023 09/01;4:100257. doi: 10.1016/j.health.2023.100257.
16. Bickmore TW, Utami D, Matsuyama R, Paasche-Orlow MK. Improving access to online health information with conversational agents: a randomized controlled experiment. *J Med Internet Res*. 2016 Jan 4;18(1):e1. PMID: 26728964. doi: 10.2196/jmir.5239.
17. Gomaa S, Posey J, Bashir B, Basu Mallick A, Vanderklok E, Schnoll M, et al. Feasibility of a text messaging-integrated and chatbot-interfaced self-management program for symptom control in patients with gastrointestinal cancer undergoing chemotherapy: pilot mixed methods study. *JMIR Form Res*. 2023 Nov 10;7:e46128. PMID: 37948108. doi: 10.2196/46128.
18. Caru M, Abdullah S, Qiu L, Kanski B, Gordon B, Truica CI, et al. Women with metastatic breast cancer don't just follow step-count trends, they exceed them: an exploratory study. *Breast Cancer Res Treat*. 2023 Jul;200(2):265-70. PMID: 37227610. doi: 10.1007/s10549-023-06980-6.
